# Developing ultrasound-assisted hot-air and infrared drying technology for sweet potatoes

**DOI:** 10.1016/j.ultsonch.2022.106047

**Published:** 2022-05-20

**Authors:** Muhammad Tayyab Rashid, Kunlun Liu, Mushtaque Ahmed Jatoi, Bushra Safdar, Dingyang Lv, Dengzhong Wei

**Affiliations:** a College of Food Science and Engineering, Henan University of Technology, Zhengzhou 450001, China; b Department of Botany, Shah Abdul Latif University, Khairpur 66020, Pakistan; c Beijing Advanced Innovation Center for Food Nutrition and Human Health, Beijing Technology & Business University, Beijing 100048, China

**Keywords:** Sweet potatoes, Drying, Ultrasound, Mathematical modeling, Exergetic analysis

## Abstract

The influence of ultrasound (US) pretreatments combined with infrared (IRD) and hot-air (HAD) drying on drying kinetics, mathematical modeling, bioactive compounds (antioxidant activities, Vitamin C, phenolics, and flavonoid contents), qualitative properties (*β*-carotene, total carotenoids, color indexes, textural profile), enzyme inactivation, and exergetic analysis of sweet potatoes. The US pretreatment at 40 kHz combined with IRD and HAD (70 °C) significantly lessened the drying time and water contents. Besides, it did not affect the sweet potato's bioactive components and other quality-related attributes. The samples’ activation energy (*E*_a_) ranged from 17.60 to 29.86 kJ/mol for both dryers, with R^2^ (0.999–0.9809). Control samples had the highest specific energy consumption (*SEC*) due to the extended drying period, whereas ultrasound (40 kHz) treated samples had the lowest *SEC* during HAD and IRD at 80 °C. The thermodynamic parameters indicated that increasing the drying temperature lowers the enthalpy and Gibbs free energy, while entropy resulted in negative values. HAD had better textural qualities (hardness and resilience). The US pretreatments followed by HAD or IRD may lead to an energy-efficient method with acceptable quality maintenance.

## Highlights

• US assisted drying with two drying techniques employed for the first time for SP.
• Hii model found fit for predicting the drying kinetics in HAD and IR.
• Lower US frequencies preserved the nutritional features in dried SP.
• The developed drying protocol helpful in energy consumption.

## 1 Introduction

Sweet potato (*Ipomoea batatas* L.) is technically a perennial vegetable vine crop, but it is typically grown as an annual due to its warm-weather requirements. Since it is a seasonal crop that must be utilized within a specific time frame after harvesting, it has a short storage period with high moisture content (MC) that encourages microbial activity, leading to deterioration. The convective hot-air drying process is frequently used for preserving sweet potatoes. It is rich in amino acids, *β*-carotene, starch, cellulose, vitamin C, and other nutrients. Sweet potatoes have medicinal benefits such as maintaining the body’s acid-base balance, preventing cancer, and lowering blood fats. Raw or processed sweet potatoes can be used in bakery products and snacks and consumed as a staple food [1]. New non-destructive technologies are always a concern for scientists as the world's population grows and non-renewable energy sources become scarcer. The factors commonly considered for implementing new technologies include minimizing energy consumption, enhancing product quality, increasing production rates, and reducing processing time [2]. Moisture reduction or drying (through simultaneous mass and heat transfer) is usually employed to extend the shelf-life of vegetables and fruits, reduce postharvest losses, and maintain quality [3]. Numerous techniques are used to dry food materials, including hot-air, solar, oven, microwaves, or infrared drying. One of the oldest and most frequently employed methods is hot-air drying which involves simultaneous heat and mass transfer and phase change, making it an energy-intensive approach [[4], [5], [6]]. The necessity of high-quality fast-dried foods leads to a renewed interest in drying operations. In addition, there is an increased demand to combine non-destructive methods with different drying technologies to boost efficiency, and minimal quality reduction has gained attention. Application of pretreatments (including ultrasonic wave, blanching, microwave) is the recent addition involving mass transfer during air drying. Wherein ultrasonic waves technique, a non-thermal approach, is recently attracting the researcher's attention as an effective pretreatment for food drying and preserving the physical and sensory features of the end-product [[3], [5], [7]]. This approach is used in a variety of ways. For example, a long-range sound wave is transmitted inside the material. The material's properties are then determined by examining the mutual effects of wavelength and substance. Integrating ultrasonic waves with other processes improves moisture diffusivity and efficiency and reduces processing time and product manufacturing costs [[8], [9], [10], [11]]. Ultrasonic pretreatments are often used with traditional drying methods like hot-air, microwave, or infrared [12]. This is accomplished by the samples immersion in a hypertonic aqueous phase or simply in water before drying, which removes moisture from the materials without significantly raising the temperature, resulting in minor damage to the food than conventional drying methods [7]. In contrast, ultrasonic waves can substantially increase the heat-mass transfer rate because of the shift in boundary layers, material diffusivity, and density [13].

Pretreatments can be combined with drying to improve the final product's quality while falling drying time and energy consumption. Using infrared-vacuum drying, Salehi & Kashaninejad [14] found significant improvements in the quality of color variables, diffusivity of moisture content, surface fluctuations or shrinkage, and lemon slices' drying kinetics. Baeghbali et al. [15] found an ultrasound infrared-assisted conductive hydro-dryer useful for apple slices drying than freeze-drying and cabinet drying techniques. Ren et al. [6] also observed the positive impact of blanching and ultrasonic pretreatments on the phenolic compounds (total flavonoid contents, total phenolic compounds, and quercetin), antioxidant capacities, and overall quality of the dried onions in comparison with the hot-air drying and lyophilization techniques.

Most such drying studies primarily focused on investigating the drying kinetics using thin-layer drying models. Even though several studies are available on the drying of sweet potatoes, as per a thorough search of the literature, no known research is available on the sequential ultrasound treated hot-air and infrared dried sweet potatoes. There is also no comparative study on the effectiveness of combination approaches like combined hot-air and infrared drying of sweet potatoes. In order to fill in the knowledge gap and contribute to the scientific literature, the objectives of this study were constructed to investigate the effects of ultrasonic pretreatments on drying kinetics, nutritional composition, enzymatic browning, textural profile, enzyme inactivation, mathematical modeling, color, and exergetic analysis of dried sweet potatoes using hot-air and infrared drying methods.

## 2 Material and methods

### 2.1 Preparation of samples

Orange flesh sweet potatoes were purchased from Zhenjiang, Jiangsu, China. A stainless-steel food slicer was used to slice the samples (thickness of 3 mm, 35 mm diameter). The samples were processed within 12 h after buying. According to the AOAC Official Method, the obtained initial moisture content was 72.80 %.

### 2.2 US pretreatment

The samples (sliced sweet potatoes) were packed using plastic bags and submerged in an ultrasonic bath (Meibo Biotechnology Co., China). Mono-ultrasound (US) frequencies, i.e., 20, 40, and 60 kHz, were employed (30 min, acoustic power 300 W/cm^3^) in a 6.0l ultrasonic tank and the water bath (30 ± 0.5 °C). A peristaltic pump monitored the water flow with a speed of 300/rpm and 50 W/L ultrasonic power density. The mono US frequency samples were inspected for 10 s *off* and 5 s *on*, then recycled back and forth.

### 2.3 Drying of sweet potato slices using different techniques

#### 2.3.1 Convective HAD

The experiment was conducted using the previously published approach with some changes [5]. Samples were dried in a hot-air drier with a 60, 70, and 80⁰C temperature range (25 % relative humidity and 1.5 m/s air velocity). Each sample (80 g) was placed in the drying chamber until a uniform weight was achieved. Samples weights were taken after 10 min of intervals until the required moisture content of 5.0 ± 1.0 % (on a wet basis) was not obtained.

#### 2.3.2 Catalytic infrared Drying. (CIR)

According to our previously reported method, the CIR dryer (Zhenjiang Maybo Innovation Co., ltd. Jiangsu, China) was employed to dry sweet potato slices [9]. The CIR emitter and samples were established with 60, 70, and 80 °C surface temperature ranges. Each sample was weighed every 10 min during drying.

### 2.4 Drying kinetics

An empirical model was employed to determine all samples' dry matter moisture ratio (MR) (Eq. (1)).

(1) $$\mathrm{MR}=\frac{M-M_{e}}{M_{0}-M_{e}}$$

where;

M = moisture content at every time intervals, *M_e_* = equilibrium water content and *M_0_* = initial water content.

Eq. (2) was used to determine the DR of all samples at a given point.

(2) $$\mathrm{DR}=\frac{M_{t1}-M_{t2}}{t_{1}-t_{2}}$$

where t_1_ and t_2_ denote the drying times (min), and *M*t_1_ and *M*t_2_ represent the samples' moisture contents (kg water/kg dry matter).

#### 2.4.1 Analysis of drying data through mathematical modeling

To characterize the drying behavior of ultrasound pretreated samples, the drying kinetics of the samples were measured using various thin layer models (Supplementary Table-S1).

#### 2.4.2 Analysis of non-linear regression

The non-linear regression analysis was performed using Origin 9.2. The excellent fit between modeling and the experimental data was estimated using four statistical functions (R^2^, RMSE, RSS, and reduced χ^2^). Higher R^2^ and lower RSS, RMSE, and χ^2^ values indicate a better model fit [16].

### 2.5 Activation energy (*E*_a_) & thermodynamics analysis

The activation energy (*E*_a_, KJ/mol) was estimated through Arrhenius Eq. (3).

(3) $$\ln\left(k\right)=\ln\left(C\right)-\frac{\mathrm{Ea}}{\mathrm{RT}}$$

where C, *E*_a_, T, and R represent the Arrhenius constant, energy activation (kJ/mol), temperature (K), and general gas-constant (8.314 J/(mol K)), respectively.

Equations (4), 5, and 6 were used to calculate thermodynamics characteristics such as entropy (ΔS), Gibbs free energy (ΔG), enthalpy (ΔH) changes based on the *E*_a_:

(4) $$\Delta S=\frac{\Delta H-\Delta G}{T}$$

(5) $$\Delta G=R\times T\times In\left(\frac{{k\times h}_{P}}{T\times K_{B}}\right)$$

(6) $$\Delta H=E_{a}-RT$$

$K_{b}$ and $h_{p}$ are Boltzmann (1.3806 10–23 J/K) and the Planck constant (0.6262–10-34 J/s).

### 2.6 Total energy consumption

An electric energy meter calculated every sample's total energy consumption (TEP) at various RH conditions calculated by the meter for electric energy (correctness of 0.01 kWh). The specific energy consumption (SEP) (the energy needed to dry 1 kg of sweet potato slices) was measured by Eq. (7) [17].

(7) $$E_{s}=\frac{E_{t}}{W_{w}}x1000$$

where$E_{s}$ = ESP, $E_{t}$=TEP, and$W_{w}$ = initial weight of samples.

### 2.7 Quality parameters

#### 2.7.1 Analysis of total phenols (TPC) and flavonoid (TFC)

The TPC (mg.GAE.g^−1^) was measured by our previously published method of dried sweet potatoes [18]. 2.5 mL of 0.2 N Folin-phenol Ciocelteu's reagent and 2 mL of 7.5 % Na_2_CO_3_ were added to a 0.5 mL extract. The mixture was incubated at room temperature in the dark for 30 min. The sample absorbance was evaluated at 765 nm using a UV-spectrophotometer (Tu-1810; Universal Instrument Purkinje Co., ltd., Beijing, China). For TFC, 1 mL of extract was put in a volumetric glass with a capacity of 10 mL. The volumetric glass was then filled with clean water until it held 5 and 0.3 mL NaNO_2_ (5 %). After 5 min, 0.3 mL AlCl_3_ (10 %) was added. After 6 min, 2 mL NaOH (1 M) was added, and the total volume of the solution was reduced to 10 mL by adding clean water. The resulting solution was thoroughly mixed, and the absorbance at 510 nm was measured. The TFC was examined by aluminum chloride assay for Rutin equivalent (mg.RU.g-1) [19].

#### 2.7.2 Vitamin-C analysis by HPLC

Vitamin-C content (mg.100.g^−1^) was evaluated using HPLC method [20]. 80 mL of 3 % *meta*-phosphoric acid solution was added to 5 g of sample, placed in a shaker for 10 min, and then the volume was made up to 100 mL using 3 % *meta*-phosphoric acid solution. Finally, the filtrate was shifted to HPLC vials. The detector’s wavelength was 254 nm.

#### 2.7.3 Total carotenoids

Total carotenoids were inspected in all samples by Tang et al. [21]. In brief, a 0.5 g sweet potato sample was extracted in triplicate using 5 mL of ethanolic butylated hydroxyl toluene (ethanol/BHT − 100:1, v/w) for carotenoid separation and release. The mixture was then mixed well before being put in a water bath at 85 °C for 5 min. After that, 0.5 mL of 80 % KOH was added for saponification and thoroughly vortexed before returning to the 85 °C water bath for 10 min. After cooling in an ice bath, the mixture was added to 3 mL of cold deionized water. *N*-Hexane (3 mL) was added to the mixture before centrifugation at 7500 rpm for 5 min to separate the two layers. The yellow top layer was transferred and collected. This treatment was repeated four times until the top layers became colorless. The samples were measured at 450 nm and 503 nm wavelengths against a blank of hexane. Total carotenoids (µg. g^−1^) were computed by following Eq. (8).

(8) $$\mathrm{Totalcarotene}=4.642\times A_{450}-3.091\times A_{503}$$

#### 2.7.4 *β*-Carotene determination

It was investigated by following the Jatoi et al. [22] method with no changes. Here, a 10 mL acetone-hexane (4:6; v/v) combination was added to a methanolic extract (100 mg sample), vortexed for 1 min, and filtered using Whatman No. 4 filter paper. The ultimate volume was determined to be 10 mL. Optical absorbance was observed at 453 nm, 505 nm, and 663 nm. The results are presented as µg.*β*-carotene g.^-1^ Fw (g.g^−1^ Fw). The following equation was used to calculate the *β*-carotene levels:

(9) $$\beta -carotene\left(mg.100{\mathrm{mL}}^{-1}\right)=0.216\times A_{663}-0.304\times A_{505}+0.452\times A_{453}$$

#### 2.7.5 Antioxidant properties

The antioxidant activities, including ABTS (mmol TE.g^−1^ dm), DPPH (µmol. TE 100 g^−1^ dm), FRAP (mmol Fe. g^−1^ dm), and reducing power (mg. g^−1^ dm), was inspected by the methods described by Rashid et al. [[9], [18], [19]].

### 2.8 Extraction of enzymes

As described by Sarpong et al. [16], the enzyme extraction was performed with no modifications. 5 g of dried sweet potato slices were powdered and combined with a 25 mL extraction solution (0.2 M phosphate buffer at pH 6.5, 1 % (v/v) Triton X-100, and 4 % polyvinyl polypyrrolidone). This was continually mixed for 3 min and maintained at 4oC for 4 h. The mixture was centrifuged for 10 min at 4 °C at 10,000 rpm, and the supernatant was collected and filtered through a 0.45 m filter membrane before being tested as crude enzyme extract.

### 2.9 Analyses of enzymes

#### 2.9.1 Polyphenol Oxidase (PPO) activity

The PPO was measured in the same way as performed by Sarpong et al. [16]. The crude enzyme extract was evaluated by adding 0.5 mL to 200 l (0.1 M) catechol and 1.5 mL to 0.1 M sodium phosphate buffer (pH 7.0). The absorbance was measured continuously at 420 nm for 5 min. The sample prepared using an enzyme-free mixture solution was termed as blank.

#### 2.9.2 Peroxidase (POD) activity

It was determined at 470 nm spectrophotometry by Zhang et al. [23] method without any modification. 0.15 mL of 4 % (v/v) guaiacol, 0.15 mL of 1 % (v/v) H_2_O_2_, 2.66 mL of 0.1 M phosphate buffer pH 7, and 40 µl of crude enzyme extract were used in the process. The solution mixture was used in the blank samples without enzyme extract.

(10) $$\mathrm{Relativeactivity}\left(\%\right)=\frac{\mathrm{CurrentEnzymeactivity}}{\mathrm{InitialEnzymesactivity}}\times 100$$

#### 2.9.3 Browning evaluations (BI)

BI of dried sweet potatoes was determined by Sarpong et al. [16], and the absorbance was monitored at 420 nm. The BI values were calculated by equations (10), (11) as described by Ruangchakpet & Sajjaanantakul et al. [24].

(11) $$\mathrm{BI}=\frac{100(x-0.31)}{0.17}$$

(12) $$X=\frac{a*+1.75L*}{5.645L*+a*-0.301b*}$$

### 2.10 Surface color

The CIE lab system was used to examine the surface color of the samples with a handheld colorimeter (DC-*P*3, Beijing, China). The overall color difference (ΔE) was measured using Eq. (13) [25]. Meanwhile hue angle (h*) Eq.14, Chroma (C*) Eq. (15), yellowness index (*YI*) Eq. (16), and whiteness index (*WI*) Eq. (17) were also evaluated, respectively.

(13) $$\Delta E={\left[{\left(L_{0}^{*}-L^{*}\right)}^{2}+{\left(a_{0}^{*}-a^{*}\right)}^{2}+{\left(b_{0}^{*}-b^{*}\right)}^{2}\right]}^{1/2}$$

(14) $$h^{*}=\tan^{-1}\left(\frac{b*}{a*}\right)$$

(15) $$C^{*}={\left[a^{*2}+\;b^{*2}\right]}^{1/2}$$

(16) $$\mathrm{YI}=\frac{142.56b*}{L*}$$

(17) $$\mathrm{WI}={\left[\left(100-L^{*2}\right)+a^{*2}+\;b^{*2}\right]}^{1/2}$$

### 2.11 Analyses of texture profiles (TPA)

The TPA of sweet potatoes was quantified by a texture analyzer (TA-XT2 of Stable MicroSystems, Ltd., Godalming, United Kingdom) equipped with a P/2N piercing plunger. The piston's speed and the stab force depth on the sample surface were 0.5 mm.sec^-1^ and 5.0 mm, respectively. The measurements were repeated four times for each sample. Hardness and resilience were assessed using Micro Stable software (Stable Micro Systems, Surrey, England).

### 2.12 Statistical study

Data are presented as mean ± SD and were checked by the Origin Pro 9.2 program. Two-way ANOVA and Tukey's test was used to compare the obtained data.

## 3 Results and discussion

### 3.1 Mathematical modeling and kinetics of drying

**Fig. 1:**
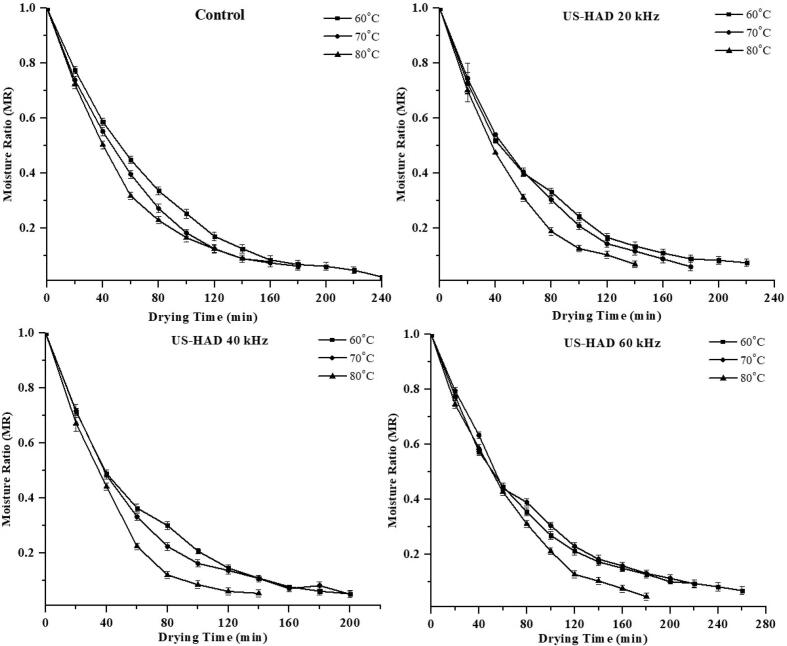
Effect of US pretreatments and HAD on moisture loss of sweet potatoes.

**Fig. 2:**
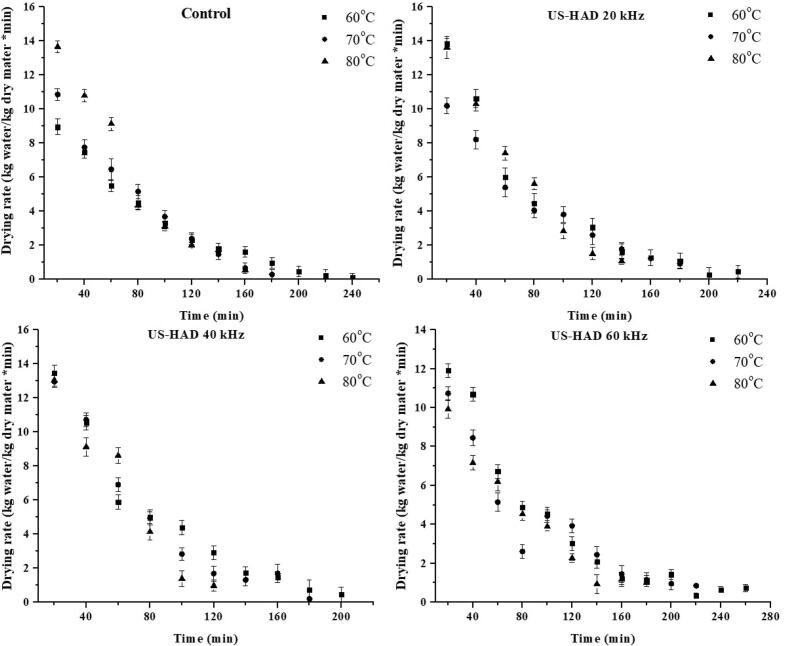
Effect of US pretreatments and HAD on drying rate of sweet potatoes.

The moisture level of sweet potato slices changed over time as a function of the experimental parameters, as shown in Fig. 1, Fig. 2. As per the results obtained, it was increasing ultrasonic intensity and drying temperatures reduced the duration of drying and moisture level. During the ultrasound-assisted drying (USAD) of sweet potato slices, the pretreated ultrasound samples lost more moisture than the control samples due to texture degradation and the lack of a firm layer. HAD of sweet potato slices took 140 min at 70 °C using ultrasound frequencies (USF) of 20 and 40 kHz, while IRD took 50 min using USF of 20 and 40 kHz. The drying time of sweet potato slices were condensed to 150 min in HAD while 50 min in infrared drying (IRD) by applying 30 min of ultrasonic pretreatment. This demonstrates that longer drying durations were needed when the sweet potato slices were not pretreated with ultrasound. It has been reported that ultrasonic waves create the cavitation phenomena, causing dramatic fluctuation (expansions/contractions) in the texture of the material and, as a result, transform the product's structure into a sponge-like tissue, allowing moisture to be removed from the product more quickly [5].

**Fig. 3:**
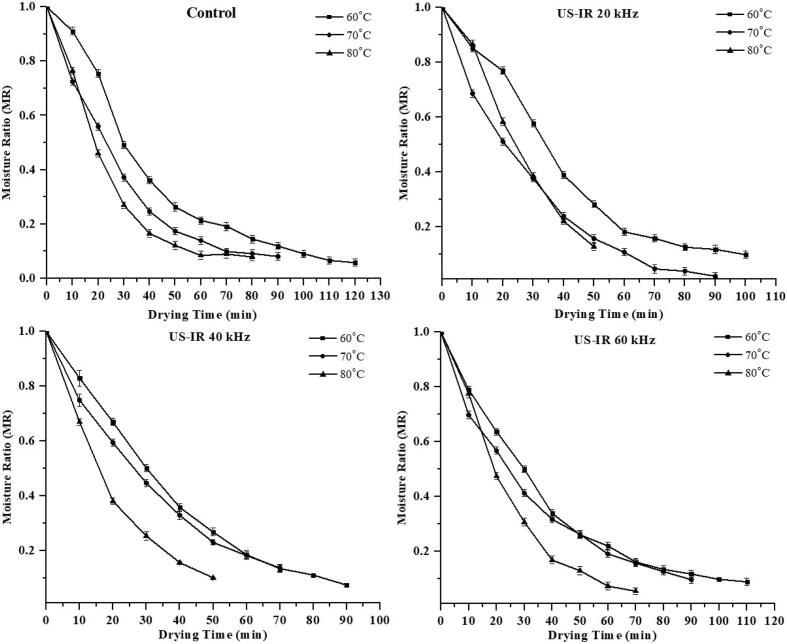
Effect of US pretreatments and IRD on moisture loss of sweet potatoes.

**Fig. 4:**
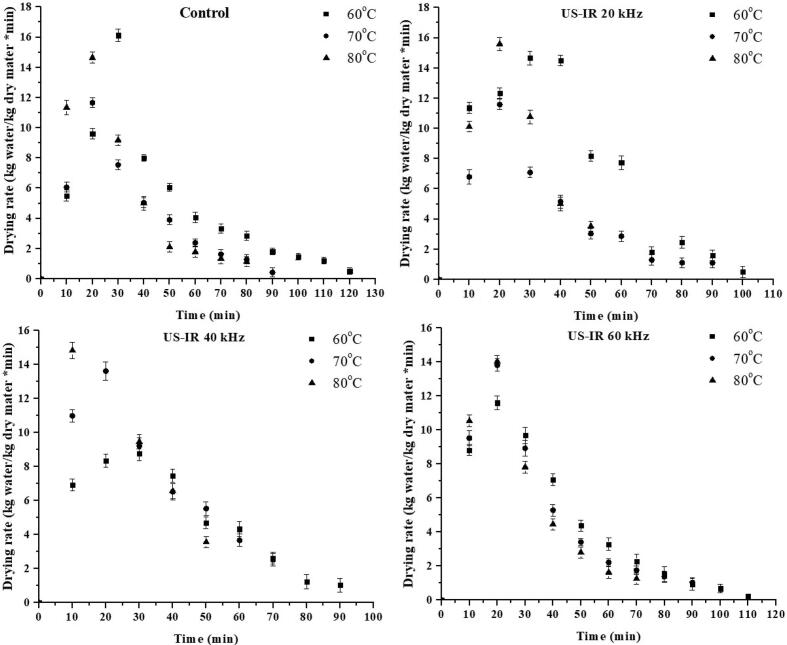
Effect of US pretreatments and IRD on drying rate of sweet potatoes.

As illustrated in Fig. 1, Fig. 2, the MC of sweet potato slices declined with elevation in temperature. The initial MC was high during the drying process, indicating a higher moisture content. This change is due to the material's internal water diffusion to the material's surface, and the MC gradually decreases with a decreasing gradient over time. The drying time was longer than at higher temperatures in the lower temperature range. Drying times reduce as the temperature rises because of the high thermal gradient, giving a greater evaporation rate. The obtained outcomes are in line with previous reports conducted for drying numerous vegetables and fruits, e.g., sweet potatoes [26], apricots [27], almonds [8], and pineapple [28], using hot-air and infrared dryers with ultrasound pretreatment. Fig. 3, Fig. 4 demonstrate the drying rate curves for pretreated ultrasonic samples dried in HAD and IR drying. It was discovered that US-treated samples had the highest drying rate throughout the drying period. Similar findings were made by Nowacka et al. [29] in dried ultrasonic pretreated apples. Initially, US pretreated samples dried in HAD and IRD (at US-40 kHz and 80 °C) had the highest drying rate, i.e., 13.05 and 16. 69 Kg/Kg dm, respectively.

Hence, the US increased the moisture removal rate during convective drying. Both the control and US pretreated samples only showed the falling-rate period using HAD, whereas the samples dried with IR showed both the regular and falling-rate periods (Fig. 3, Fig. 4). This stage ended after about 20–30 min of drying. When the samples were ultrasonically pretreated before convective drying, they may have taken up water. Water on the sample surface was loosely bound, causing the IR drying constant rate. The falling rate of the samples indicates that the drying approach is regulated by diffusion. Comparable findings were reported for cocoyam [30], sweet potato [9], corn, and wheat [31]. Sweet potato drying models were evaluated using the experimental data. Table 1, Table 2 show the non-linear regression applied to the empirical data statistical modeling.

**Table 1:** Averages of selected models fitted to thin-layer drying using the US and HAD for sweet potatoes.

Sample Codes	T (°C)	Coefficients	R^2^	RSS	χ^2^	RMSE
**Hii Model**						
CTL1	60 70 80 60 70 80 60 70 80 60 70 80	a. 0.652, k1. 0.010, n. 1.080, b. 0.346, k2. 0.010 a. 0.030, k1. 0.888, n. 1.119, b. 0.970, k2. 0.009 a. 0.831, k1. 0.010, n. 1.204, b. 0.169, k2. 0.002 a. 1.001, k1. 0.021, n. 0.920, b. 4.94 × 10^-6^, k2. −0.060 a. 1.000, k1. 0.014, n. 1.029, b. 1.83 × 10^-5^, k2. −0.024 a. 0.996, k1. 0.012, n. 1.123, b. 0.003, k2. −0.009 a. 0.679, k1. 0.022, n. 0.934, b. 0.323, k2. 0.022 a. 0.695, k1. 0.011, n. 1.222, b. 0.305, k2. 0.003 a. 0.997, k1. 0.010, n. 1.226, b. 0.000, k2. −0.016 a. 0.758, k1. 0.013, n. 1.079, b. 0.243, k2. 0.003 a. 0.591, k1. 0.010, n. 1.164, b. 0.409, k2. 0.003 a. 0.664, k1. 0.009, n. 1.126, b. 0.327, k2. 0.009	0.999 0.999 1.000 0.999 1.000 1.000 0.999 1.000 0.998 1.000 0.998 0.998	0.00 0.00 0.00 0.00 0.00 0.00 0.09 0.04 0.07 0.00 0.00 0.00	6.16 × 10^-5^ 5.65 × 10^-5^ 7.31 × 10^-5^ 0.0001 9.38 × 10^-5^ 2.42 × 10^-5^ 0.0001 3.33 × 10^-5^ 0.00028 4.34 × 10^-5^ 0.00028 0.00026	0.0007 0.0071 0.0069 0.0101 0.0081 0.0057 0.0122 0.0057 0.0128 0.0058 0.0137 0.0126
CTL2						
CTL3						
US-HAD1						
US-HAD2						
US-HAD3						
US-HAD4						
US-HAD5						
US-HAD6						
US-HAD7						
US-HAD8						
US-HAD9						
**Two-term model**						
CTL1	60 70 80 60 70 80 60 70 80 60 70 80	a. 13.61, k1. 0.10, b. −12.60, k2. 0.010 a. 14.12, k1. 0.01, b. −13.11, k2. 0.012 a. 1.983, k1. 0.00, b. 1.984, k2. 0.827 a. 2.205, k1. 0.00, b. 2.206, k2. 0.953 a. 5.757, k1. 0.01, b. −4.75, k2. 0.012 a. 1.878, k1. 0.01, b. 1.879, k2. 0.775 a. 2.058, k1. 0.00, b. 2.059, k2.0.934 a. 1.004, k1. 0.01, b. 0.003, k2. −0.014 a. 20.91, k1. 0.015, b. −19.9, k2. 0.014 a. 0.940, k1. 0.01, b. 0.065, k2. 0.001 a. 1.001, k1. 0.01, b. 0.006, k2. −0.008 a. 11.82, k1. 0.00, b. −10.82, k2. 0.009	0.999 0.999 0.998 0.998 1.000 0.998 0.999 0.999 0.996 1.000 0.997 0.998	0.00 0.00 0.00 0.00 0.00 0.00 0.09 0.15 0.10 0.00 0.00 0.00	6.90 × 10^-5^ 9.90 × 10^-5^ 0.00023 0.00018 9.40 × 10^-5^ 7.30 × 10^-5^ 0.00018 0.00067 0.00067 5.30 × 10^-5^ 0.00028 0.00019	0.0076 0.0094 0.0122 0.0129 0.0081 0.0100 0.0122 0.0257 0.0195 0.0064 0.0152 0.0107
CTL2						
CTL3						
US-HAD1						
US-HAD2						
US-HAD3						
US-HAD4						
US-HAD5						
US-HAD6						
US-HAD7						
US-HAD8						
US-HAD9						

CTL = Control; US-HAD = Ultrasound hot air drying.

**Table 2:** Average statistical parameters of selected models fitted to thin-layer drying using the US and IRD for sweet potatoes.

Sample Codes	T (°C)	Coefficients	R^2^	RSS	χ^2^	RMSE
Hii Model						
CR1	60 70 80 60 70 80 60 70 80 60 70 80	a. 0.674, k1. 0.00, b. 0.319, k2. 4.50 × 10^-5^n. 2.21 a. 0.993, k1. 0.02, b. 0.003, k2. −0.019, n. 1.11 a. 0.745, k1. 0.00, b. 0.255, k2. 0.001, n. 1.63 a. 2.975, k1. 0.00, b. 2.979, k2. −3.63 × 10^-5^n 177. a. 0.678, k1. 0.02, b. 0.313, k2. 0.028, n. 1.062 a. 0.364, k1. 0.00, b. 0.637, k2. 0.000, n. 2.107 a. 0.992, k1. 0.00, b. 0.004, k2. −0.005, n. 1.284 a. 0.717, k1. 0.02, b. 0.279, k2. 0.024, n. 1.043 a. 0.713, k1. 0.02, b. 0.287, k2. 0.026, n. 1.195 a. 0.024, k1. 0.00, b. 0.972, k2. 0.015, n. 1.159 a. 0.920, k1. 0.02, b. 0.080, k2. 2.391, n. 0.976 a. 0.583, k1. 0.00, b. 0.418, k2.0.002, n. 1.644	0.999 0.998 1.000 0.996 0.996 1.000 0.999 0.999 0.996 0.998 0.999 0.999	0.00 0.00 0.00 0.00 0.00 0.00 0.00 0.00 0.00 0.00 0.00 0.00	0.00015 0.00016 1.40 × 10^-5^ 0.00039 0.00027 2.20 × 10^-5^ 6.87 × 10^-5^ 8.90 × 10^-5^ 0.00061 0.00017 0.00014 0.00010	0.1124 0.0118 0.0034 0.0196 0.0154 0.0058 0.0078 0.0100 0.0201 0.0123 0.0111 0.0092
CR2						
CR3						
US-IR1						
US-IR2						
US-IR3						
US-IR4						
US-IR5						
US-IR6						
US-IR7						
US-IR8						
US-IR9						
**Page Model**						
CR1	60 70 80 60 70 80 60 70 80 60 70 80	k. 0.009, n. 1.245 k. 0.030, n. 1.026 k. 0.017, n. 1.250 k. 0.006, n. 1.351 k. 0.030, n. 1.051 k. 0.005, n. 1.566 k. 0.010, n. 1.234 k. 0.024, n. 1.036 k. 0.024, n. 1.218 k. 0.022, n. 1.028 k. 0.044, n. 0.088 k. 0.016. n. 1.272	0.986 0.996 0.995 0.989 0.996 0.999 0.999 0.999 0.996 0.996 0.999 0.997	0.02 0.00 0.00 0.01 0.00 0.00 0.06 0.00 0.00 0.00 0.00 0.00	0.00145 0.00025 0.00061 0.00108 0.00044 9.2 × 10^-5^ 6.06 × 10^-5^ 8.90 × 10^-5^ 0.00046 0.00027 0.00016 0.00033	0.0350 0.0149 0.0227 0.0327 0.0195 0.0120 0.0073 0.0100 0.0174 0.0156 0.0121 0.01692
CR2						
CR3						
US-IR1						
US-IR2						
US-IR3						
US-IR4						
US-IR5						
US-IR6						
US-IR7						
US-IR8						
US-IR9						

CR = Control; US-IR = Ultrasound infrared drying.

**Fig. 5:**
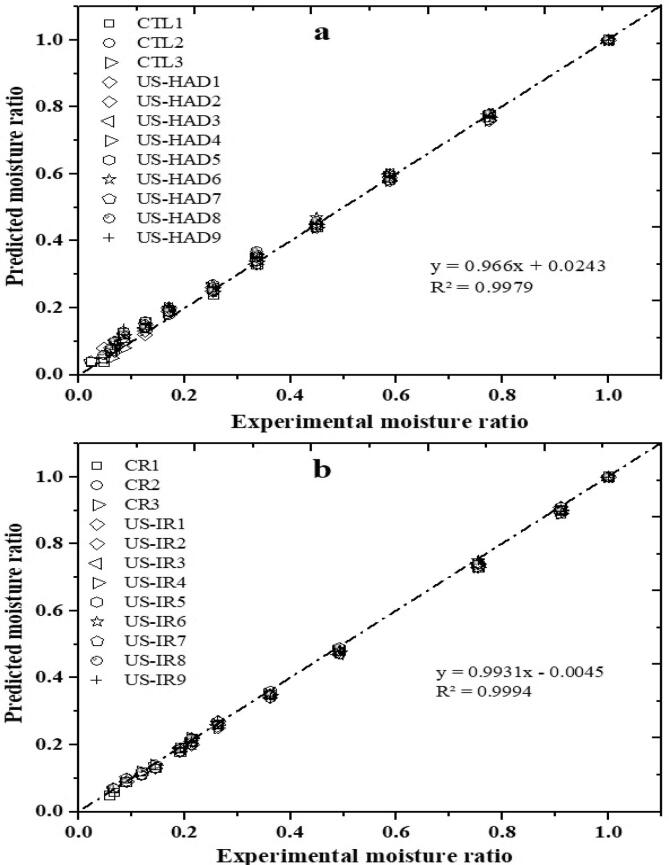
Comparison of experimental vs predicted moisture ratio of Hii model for HAD and IRD dryers.

There were four metrics used to evaluate models: R^2^ (coefficient of determination), reduced chi-square (χ2), residual sum of squares (RSS), and root mean square error (RMSE). The Hii model was selected for sweet potato drying characteristics as it presented uppermost R^2^ and lowest RSS (χ^2^) and RMSE. This model gave the most acceptable representation of sweet potato slices' drying characteristics in both HAD and IRD dryers for pretreated ultrasound samples (Table 1, Table 2). Fig. 5 compares experimental MR for various samples at a drying temperature of 60, 70, and 80 °C. This trend provides vital support to the models' ability to predict the drying characteristics of sweet potatoes. Hii et al. [32] and Fan et al. [33] described the Hii model's accuracy in drying cocoa and sweet potato slices, respectively, with higher R^2^, lower RSS, χ^2^, and RMSE. The Two-term equation was the second best-fitting model in HAD after the Hii model. Dash et al. [34] made a similar discovery regarding the predictive ability of a two-term exponential model for star fruit drying performance.

On the other hand, the Page model fits the drying characteristics of ultrasound pretreated and control samples in IRD drying better than any different model. The Page model successfully predicted fresh cashew drying in a fixed bed dryer and can relate to the current study [[35], [36]]. Whereas the other tested models were found unsuitable and suitable, tabulated in supplementary Tables S2 and S3 just for comparison.

### 3.2 Exergetic analysis

#### 3.2.1 *E*_a_ analysis

The activation energy, or Ea, is the smallest energy required to overcome an energy barrier and start a process. The *E*_a_ values ranged between 17.60 and 29.86 kJ/mol with an R^2^ value of 0.999–0.9809. (Table-3). The investigation results fall within 11.57–36.44 kJ/mol, as stated by Onwude et al. [3], and 17.40–33.94 kJ/mol reported by Doymaz [37]. The samples recorded at 20 kHz and 40 kHz had the highest and lowest *E*_a_ in HAD and IRD dryers. The lower the *E*_a_, the less energy is needed to achieve the active state. It accelerated mass transfer and diffusion by causing the products to dry more quickly (higher moisture effective diffusivity). Other studies have found a similar pattern [[16], [27]]. Pretreatments, material type, and temperature affect activation energy [38].

#### 3.2.2 Thermodynamics

**Fig. 6:**
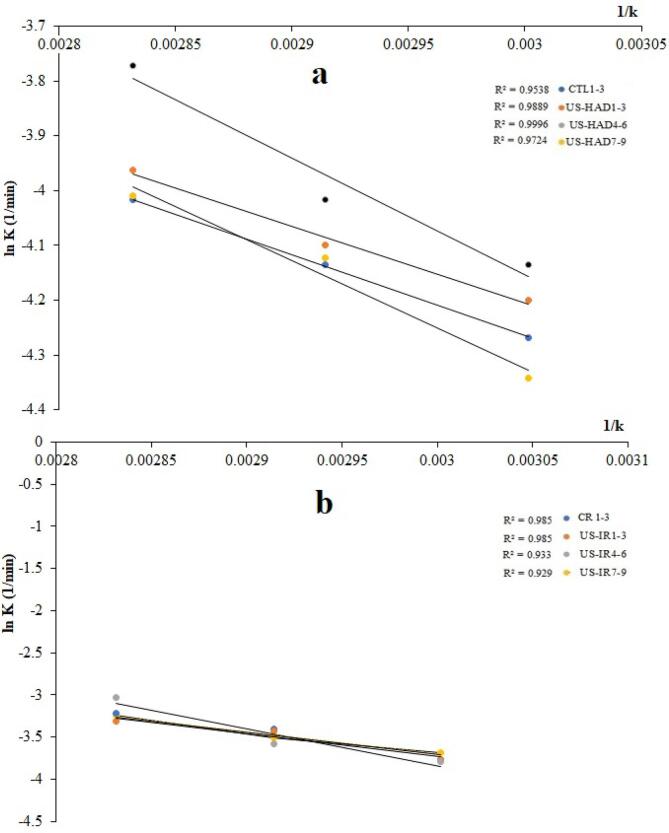
The natural logarithm (ln vs slope 1/k) represents sweet potatoes' activation energy (Ea) in HAD and IRD drying.

Table 3 shows the thermodynamic functions (enthalpy, Gibbs, and entropy), demonstrating the minimum energy necessary to remove a certain amount of water. The natural logarithm of the rate constant vs 1/*K* is evident from the Arrhenius plots (Fig. 6a & b), implying that the slope of the plots indicated the hot-air and infrared drying dynamics. The enthalpy shifts (ΔH) (stated as the discrepancy in the reactant and the activated complex energy) were calculated using Eq (4). According to Table 3, ΔH values determined for HAD and IRD drying were similar, ranging from 8.60 to 14.91 kJ/mol for HAD and 18.77 to 33.77 kJ/mol for IRD dryers, respectively. Almost similar values were noted in dried banana slices ranging from 9.49 to 13.59 kJ/mol [16]. According to Eq. (4), temperature fluctuation had minimal influence on ΔH because of the less quantity of the ideal gas constant (R). As a result, differences in ΔH values were mainly attributable to variations in ultrasonic pretreatments, resulting in a direct relationship between ΔH and US. After ultrasonic pretreatment of sweet potato slices, the specific enthalpy of the drying process increased. Both dryers had decreased enthalpy values at 20 kHz US frequency, demonstrating the influence of low-frequency US pretreatments. However, the enthalpy was reduced when the drying temperature and US frequency were increased to 80 °C and 60 kHz. Furthermore, the enthalpy values were positive, consistent with Rashid et al. [9] findings for drying sweet potatoes. This finding supports the existence of endothermic reactions and the required heat to influence physical and chemical changes in vacuum-dried apples [39].

**Table 3:** Effect of US pretreatments with HAD and IRD drying methods on sweet potatoes regarding activation energy, thermodynamics, and specific energy consumption.

Ultrasonic Frequency (kHz)	Sample codes	Temperature (^o^C)	Activation Energy (kJ/mol)	R^2^	ΔH(kJ/mol)	ΔG(kJ/mol)	ΔS(kJ/mol)	Energy Consumption (kWh/kg)
**HAD**								
**Untreated**	CTL1 CTL2 CTL3	60 70 80	12.30	0.9996	12.92 12.84 12.76	185.44 190.71 196.01	−517.84 −518.35 −518.91	14.08 10.56 9.39
**20**	US-HAD1 US-HAD2 US-HAD3	60 70 80	11.54	0.9889	8.77 8.69 8.60	185.25 190.90 195.85	−529.73 −530.99 −530.21	12.91 10.56 8.21
**40**	US-HAD4 US-HAD5 US-HAD6	60 70 80	17.68	0.9538	14.91 14.82 14.74	185.07 190.38 195.29	−510.78 −511.59 −511.25	11.73 9.39 7.04
**60**	US-HAD7 US-HAD8 US-HAD9	60 70 80	16.32	0.9724	13.55 13.47 13.38	185.65 191.53 196.54	−516.57 −518.91 −518.64	15.25 12.91 10.56
**IRD drying**								
**Untreated**	CR1 CR2 CR3	60 70 80	27.13	0.9754	24.37 24.28 24.20	184.07 188.65 193.66	−479.36 −478.98 −479.86	7.04 5.28 3.52
**20**	US-IRR1 US-IR2 US-IR3	60 70 80	21.70	0.9291	19.88 19.79 19.71	184.07 188.40 194.23	−492.84 −491.34 −494.17	5.87 4.11 3.52
**40**	US-IR4 US-IR5 US-IR6	60 70 80	36.62	0.9330	33.85 33.77 33.69	183.84 189.11 193.13	−450.19 −452.70 −451.48	5.28 4.11 2.93
**60**	US-IR7 US-IR8 US-IR9	60 70 80	22.65	0.9850	18.93 18.85 18.77	183.84 189.11 193.74	−494.98 −496.18 −495.46	6.45 5.87 3.52

CTL = Control; US-HAD = Ultrasound hot air drying. CR = Control; US-IR = Ultrasound infrared drying.

Gibb's free energy is a system activity unit in the adsorption practice that aids in the insight of the thermodynamic handling factors affecting the operations [39]. The ΔG values for HAD and IRD dryers ranged from 185.07 to 196.54 and 183.84 to 194.23 kJ/mol, respectively, indicating non-spontaneous reactions. The proximity of these outcomes suggests the rise in the system's total energy as the reagents approached. Gibb's free energy rose when the temperature increased from 60 °C to 80 °C in ultrasonic pretreated and control samples in both dryers. Drying is an endergonic reaction because it entails heat from the mediums, as shown by the positive Gibbs free energy values. One advantage of ultrasound-assisted treatments is that less heat is required to dehydrate the body's moisture. Nadi et al. [39] observed similar findings during the vacuum drying of apples.

Entropy is commonly expressed as the degree of disordering. This context is the level of disordering between moisture and the product. Entropy change (ΔS) for deterioration in sweet potato slices was used to calculate the disorder change (Table 3). The modulus entropy of the sonicated samples decreased in the current study. Similarly, a decrease in entropy has also been reported in vacuum dried apples [39].

On the other hand, this phenomenon indicates that cell disruption confers an exemplary structure of the food material, implying that moisture can travel and generate zones of elevated moisture level. This reorganization may enhance the moisture gradients and the diffusion. As a result, the product dehydrates more easily. Additionally, rising temperature for drying augmented the entropy of the process. Similar effects were noticed by Rashid et al. [9] in this temperature range.

#### 3.2.3 Consumption of specific energy

The quantity of *SEC* based on several treatments (ultrasonic pretreatments and varied temperatures) was used to compute by Eq. (19), and the results are tabulated in Table 3. The rise in temperature from 60 to 80 °C in HAD and IRD drying reduces the energy consumption in all ultrasonic pretreatments. Increasing the drying temperature of agricultural products reduces energy consumption [8]. There's also a clear correlation between increasing ultrasonic frequency and decreasing power consumption, as shown in Table 3. This is because ultrasonic waves do not form complex surface layers, which allows them to remove moisture from product tissues more quickly. Consequently, the drying process takes place in a short time and consumes less energy. The highest energy (14.08 kW/kg) was utilized at 60 °C, and the control treatment (CTL1) was in HAD.

Meanwhile, the lowest energy (7.04 kW/kg) was noted in the US-HAD6 treatment at 80 °C with a US frequency of 40 kHz. However, the control treatment consumed the most energy, i.e., CR1 (7.04) with infrared drying, and the lowest was noted in US-IR6 (2.93) at 40 kHz and a temperature of 80 °C. Santos et al. [40] also observed that as compared to Control, the energy was reduced by up to 53 % (Ethanol), 62 % (Ethanol + the US), and 41 % (Water + the US) for the ultrasound pretreatments used. These results confirmed the drying time and rate, as shown in Fig. 1, Fig. 2.

### 3.3 Effect of ultrasound and drying methods on phytochemical compounds

#### 3.3.1 Total phenolic compounds (TPC)

Table 4 depicts the differences in phytochemical components of dried sweet potato slices caused by various HAD and IRD drying combination techniques at three different temperatures (60, 70, and 80 °C) and US frequencies (20, 40, and 60 kHz). After drying, TPC, TFC, and Vit-C levels significantly increased. In HAD, TPCs of fresh, HAD, and IRD samples ranged from 3.834 to 11.267 and 1.91,7.85–18.95 mg.GAE. 100.g^−1^ dm, respectively. TPC content of dried samples was significantly (p < 0.05) elevated compared to fresh sweet potato samples. Likewise, Yang et al. [41] found a significant increase in the TPC of dried sweet potatoes. The highest TPC values i.e.,11.267 and 18.95 267 mg.GAE. 100.g-1 dm were obtained in the samples dried by US-HAD5 and US-IR5 (70 °C and 40 kHz USF), respectively, which could be ascribed to the homogeneous distribution and redistribution of temperature during the drying process. Similarly, ANOVA revealed a significant difference in TPC values among US-HAD, US-IRD, and control samples (*p < 0.05*). Due to the high temperature, TPC significantly decreased when phytochemical components were lost at 80 °C and USF 60 kHz. Thus, the lowest TPC values, i.e., 3.834 and 7.85 mg.GAE. 100.g^−1^ dm were obtained in US-HAD9, and US-IR9 dried samples. This could be due to increased polyphenol breakdown from browning and oxidation reactions caused by prolonged exposure to hot air and infrared radiation. According to An et al. [42], electromagnetic radiation produces high and rapid heat, resulting in significant thermal degradation of phenolic compounds. Consequently, the most efficient temperature was 70 °C with a US frequency of 40 kHz. Maleki et al. (2020) [11] studied the drying kinetics of carrot snacks employing US pretreatments supports our findings.

**Table 4:** Effect of US pretreatments with HAD and IRD drying methods on the phytochemical composition of sweet potatoes.

Sample codes	Temperature ^o^C	Frequency kHz	TPC mg GAE.g^−1^.dm	TFC mg GAE.g^−1^.dm	Vitamin C mg 100.^-1^ g dm	Browning Index	TCC mg 100.^-1^dm	β-carotene µ.g^−1^ dm
**HAD**								
Fresh	–	–	1.91 ± 0.14	1.07 ± 0.34	17.23 ± 0.54	0.17 ± 0.034	1.60 ± 0.02	0.86 ± 0.01
CTL1	60	–	8.912 ± 0.24f	7.70 ± 0.17c	11.67 ± 0.23j	0.28 ± 0.23g	1.16 ± 0.07k	3.17 ± 0.15l
CTL2	70	–	5.827 ± 0.25j	4.23 ± 0.14g	11.72 ± 0.22i	0.27 ± 0.24h	1.46 ± 0.07j	3.45 ± 0.10k
CTL3	80	–	8.190 ± 0.00g	4.32 ± 0.02f	11.51 ± 0.24k	0.22 ± 0.16j	2.20 ± 0.07g	4.31 ± 0.08d
US-HAD1	60	20	9.952 ± 0.24c	10.19 ± 0.01b	14.21 ± 0.21d	0.26 ± 0.25i	4.73 ± 0.21b	3.53 ± 0.17j
US-HAD2	70	20	4.816 ± 0.24k	5.77 ± 0.53d	13.95 ± 0.24e	0.38 ± 0.23d	3.30 ± 0.02e	4.27 ± 0.15e
US-HAD3	80	20	9.123 ± 0.02e	4.03 ± 0.09j	13.73 ± 0.24f	0.36 ± 0.12f	1.64 ± 0.08i	4.38 ± 0.19b
US-HAD4	60	40	9.982 ± 0.24b	4.20 ± 0.44h	15.11 ± 0.21b	0.18 ± 0.18k	3.83 ± 0.09d	4.08 ± 0.11g
US-HAD5	70	40	11.267 ± 0.08a	14.19 ± 0.29a	16.13 ± 0.20a	0.18 ± 0.17l	5.74 ± 0.10a	3.80 ± 0.14h
US-HAD6	80	40	9.139 ± 0.01d	4.10 ± 0.17i	14.11 ± 0.25d	0.37 ± 0.12e	4.36 ± 0.07c	5.19 ± 0.21a
US-HAD7	60	60	7.565 ± 0.12h	5.54 ± 0.06e	12.08 ± 0.26g	0.42 ± 0.27c	2.40 ± 0.14f	3.68 ± 0.13i
US-HAD8	70	60	6.765 ± 0.15i	3.96 ± 0.02k	11.80 ± 0.27h	0.45 ± 0.07b	1.95 ± 0.10h	4.33 ± 0.14c
US-HAD9	80	60	3.834 ± 0.24l	2.76 ± 0.05l	10.63 ± 0.20l	0.57 ± 0.06a	0.91 ± 0.07l	4.15 ± 0.23f
**IRD drying**								
CR1	60	–	8.53 ± 0.27i	5.10 ± 0.22l	11.13 ± 0.25g	0.28 ± 0.24k	3.08 ± 0.31h	2.04 ± 0.18l
CR2	70	–	8.46 ± 0.25j	6.79 ± 0.24j	12.20 ± 0.18b	0.33 ± 0.21i	3.95 ± 0.34d	3.34 ± 0.270g
CR3	80	–	10.10 ± 0.25g	5.91 ± 0.22k	7.01 ± 0.20l	0.53 ± 0.28c	3.32 ± 0.26f	3.05 ± 0.29j
US-IR1	60	20	11.90 ± 0.25d	8.20 ± 0.21f	11.53 ± 0.24e	0.48 ± 0.33f	2.94 ± 0.08j	3.46 ± 0.28c
US-IR2	70	20	9.08 ± 0.43h	7.36 ± 0.21h	12.18 ± 0.24c	0.39 ± 0.23h	3.01 ± 0.11i	2.86 ± 0.27k
US-IR3	80	20	16.08 ± 0.38b	9.47 ± 0.24c	10.92 ± 0.20h	0.52 ± 0.28e	2.54 ± 0.10k	3.14 ± 0.26i
US-IR4	60	40	10.12 ± 0.27f	9.50 ± 0.27b	12.00 ± 0.21d	0.53 ± 0.27d	5.45 ± 0.07b	3.20 ± 0.31h
US-IR5	70	40	18.95 ± 0.46a	10.15 ± 0.22a	12.20 ± 0.24a	0.19 ± 0.15l	6.48 ± 0.12a	3.59 ± 0.26b
US-IR6	80	40	13.05 ± 0.26c	8.64 ± 0.05d	11.29 ± 0.23f	0.32 ± 0.27j	4.69 ± 0.07c	3.92 ± 0.32a
US-IR7	60	60	10.44 ± 0.26e	7.61 ± 0.22g	9.80 ± 0.23j	0.43 ± 0.27g	3.76 ± 0.31e	3.45 ± 0.28d
US-IR8	70	60	8.18 ± 0.65k	8.61 ± 0.26e	9.96 ± 0.22i	0.54 ± 0.29b	3.09 ± 0.13g	3.36 ± 0.27f
US-IR9	80	60	7.85 ± 0.25l	7.27 ± 0.21i	9.69 ± 0.23k	0.55 ± 0.26a	2.49 ± 0.08l	3.44 ± 0.26e
**Temperature T)**	–	–	<0.0001***	<0.0001***	<0.0001***	<0.0001***	<0.0001***	<0.0001***
**Frequency (F)**	–	–	<0.0001***	<0.0001***	<0.0001***	<0.0001***	<0.0001***	<0.0001***
**T × F**	–	–	<0.0001***	<0.0001***	<0.0001***	<0.0001***	<0.0001***	<0.0001***

Means followed by different letters within columns are significantly different (n.s., *, ***, nonsignificant, or significant at p < 0.05, or p < 0.001, respectively).

#### 3.3.2 Total flavonoid compounds (TFC)

TFCs increased significantly in all combined methods for US-HAD and US-IRD dried sweet potato slices (Table 4). TFC of fresh sweet potatoes was 1.07. (mg.GAE. 100.g^−1^ dm). TFCs of US-HAD and US-IR dried samples ranged from 2.76 to 14.19 and 7.27–10.15 mg.GAE. 100.g^−1^ dm, respectively, was higher than fresh samples (1.07 mg.GAE. 100.g-1 dm). TFC values were found. The US-HAD5 and US-IR5 samples dried at 70 °C (USF 40 kHz) presented the highest TFC values, 14.19 and 10.15 mg.GAE. 100.g^−1^ dm, respectively. The increase in flavonoid content caused by US pretreatments may be attributed to acoustic cavitation, retaining the mechanical stress on the samples, and allowing discharge of additional flavonoid compounds [43]. The results agreed with Ren et al. [6], who discovered that ultrasonic treatment of onions increased TFC levels. The intermittent combination of US-HAD and US-IRD would cause cellular compounds to break down, making flavonoids easily accessible during extraction [42]. Both IRD and HAD had a promising effect on TFC when samples were dried at 80 °C (*p < 0.05*). Flavonoid retention was increased due to the shorter drying times, temperature effect, and less heating used in US-HAD9 and US-IR9. Similarly, results were obtained when Chinese ginger was microwaved intermittently and convectively dried [42].

#### 3.3.3 Vitamin C

The effect of the drying process and US treatments on the Vit-C content of dried sweet potato slices is shown in Table 4. All the dried samples had a significant (*p < 0.05*) reduction in vitamin C (ranging from 7.01 to 16.13 mg 100 g^−1^ dm) except fresh sample. The HAD (US-HAD5) and IRD (US-IR5) dried samples (70 °C and USF 40 kHz) maintained the highest vit-C values (16.13 and 12.20 mg 100^-1^ g dm, respectively). Ascorbic acid is well known for being relatively vulnerable to oxygen, light, and heat [44]. The Vit-C content in the US pretreated samples was comparable with the control sample (p < 0.05), indicating that US pretreatment had no beneficial effect on vit-C content due to its instability during processing of food material and susceptibility to decomposition by the influence of influence various factors including heat and light. Ercan & Soysal [45] found no significant difference in Vit-C content in the US treated samples and control. However, US-treated samples at 80 °C and 60 kHz frequency dried using both dryers retained significantly more vitamin C than those not treated. The reason could be that the cell wall was disrupted because of the alternative compression and expansion force on the samples because of ultrasound application [46]. The concentration of ascorbic acid in IRD samples was also significantly lesser than in HAD samples. Due to the inconsistency of the heating temperature during IRD drying speeds up the Vit-C oxidation, resulting in a frequent loss in it. As a result of the oxidation caused by heating, the infrared-drying temperatures negatively affected ascorbic acid retention. By combining US-treatment with IRD drying, Baeghbali et al. [15] noticed that infrared heating damaged more Vit-C in the apple slices than other procedures.

#### 3.3.4 Total carotenoids contents (TCC)

The TCC of samples following various treatments and drying processes are presented in Table 4. Based on the findings, the US pretreatments with both used techniques have a significant impact on the deterioration of TCC. The retention of carotenoids in the current investigation altered from 0.91 to 6.48 mg 100.g^−1^ dm in control and other samples processed with ultrasonic in both drying methods. Samples treated at 40 kHz by the US had higher total carotenoid content than untreated samples. This phenomenon might be associated with electroporation and sonication phenomena, resulting in more polar carotenoids (e.g., xanthophylls) leaking into the water treatment [47].

Furthermore, the degradation level of TCC was found to be dependent on the water activity of the samples, ranging between 0.5 and 0.6 mg 100.g^−1^ dm. Sonication caused partial water evaporation, particularly on the samples. Hence, the moisture content of such pre-dried samples was reduced. Increased retention of sweet potatoes pretreated before drying in the US may also be related to improved extractability of the carotenoids extraction from shattered chromoplasts [48] that changes the cell wall configuration [43]. The statistical assessment revealed that the type of US had the most significant effect on the variability of carotene retention.

#### 3.3.5 *β*-carotene

Compared to fresh samples, the concentration of *β*-carotene increased after US pretreatment in all treatments, including controls (Table 4). At 80 °C, the significantly highest *β*-carotene concentration was found in US-HAD6 (5.19 µ. g^−1^ dm), followed by US-HAD3 (4.38). A similar trend was seen in infrared drying, with the highest *β*-carotene concentration (3.92) obtained in US-IR6 at 80 °C. Wang et al. [49] discovered similar results for carrot samples, with carotene content increasing by 5.46 %, 13.07 %, and 17.62 % with ultrasonic treatment at 20 kHz. It was discovered that a shorter drying period at 80 °C preserved more carotenoids.

Additionally, it was found that the shorter drying period lowers the carotenoid loss in various varieties of sweet potatoes [50]. According to Wang et al. [49], *β*-carotene was discovered in the chromoplasts of carrot slices and was stabilized by lipoproteins. As a result, the increased *β*-carotene concentration was more than likely caused by ultrasonic extraction. The use of US on the sweet potato samples may have harmed the cellular structure, which was necessary for extracting carotene from the sample tissue during measurement.

### 3.4 Antioxidants assays

Table 5 shows the antioxidant activity of sweet potatoes in terms of ABTS, DPPH, FRAP, and reducing power. The ABTS values varied between 0.38 and 3.24 mmol TE.g^−1^ dm for the US treated and untreated samples in both dryers. The US-HAD1, US-HAD4, US-IR1, US-IR1 (20 and 40 USF and 60 °C) exhibited the highest antioxidant activity (*p < 0.05*), i.e., 2.37, 1.57, 3.24, and 2.69 mmol TE.g^−1^ dm, respectively, in terms of radical inhibition as compared to control samples. However, US-HAD9 and US-IR9 (at 80 °C and 60 kHz) presented the lowest same antioxidant activity value of 0.38 mmol TE.g^−1^ dm 80 °C. In the DPPH assay, a similar trend was observed for US-treated samples. The US-HAD1, US-HAD4, US-IR1, and US-IR4 displayed the highest DPPH antioxidant activity values, 10.26, 8.46,7.39, and 5.25 µmol TE 100.g^−1^ dm, respectively. The US-HAD9 and US-IR9 showed the lowest DPPH assay values, i.e., 1.00 and 1.50 mol TE 100.g^−1^ dm, respectively. In FRAP antioxidant activity, The highest US-HAD1US-IR1 (20 kHz and 60 °C) obtained the most elevated values of 2.45 and 2.76 mmol Fe.g^−1^ dm, respectively. However, the US-treated samples (60 kHz and 80 °C) had the lowest FRAP values of 1.17 mmol Fe.g^−1^ dm. The control samples exhibit lower antioxidant values than ultrasound-treated samples in all treatments. Similar results revealed that sonicated blackberries samples present stronger ABTS and FRAP values than hot air-dried control samples [51].

**Table 5:** Effect of US pretreatments with HAD and IRD drying methods on antioxidants assays of sweet potatoes.

Sample codes	Temperature ^o^C	Frequency kHz	ABTS mmol TE.g^−1^ dm	DPPH µmol. TE 100g^−1^ dm	FRAP mmol Fe.g^−1^ dm	Reducing power mg.g^−1^ dm
**HAD**						
CTL1	60	–	1.01 ± 0.01g	1.58 ± 0.00h	2.00 ± 0.02c	1.94 ± 0.59g
CTL2	70	–	1.31 ± 0.04c	1.28 ± 0.02k	1.77 ± 0.23g	1.59 ± 0.42k
CTL3	80	–	1.13 ± 0.01e	4.54 ± 0.02g	1.92 ± 0.21e	1.90 ± 0.47h
US-HAD1	60	20	2.37 ± 0.08a	10.26 ± 0.71a	2.45 ± 0.26a	3.13 ± 0.20a
US-HAD2	70	20	1.31 ± 0.01d	7.10 ± 0.20d	1.19 ± 0.21k	1.62 ± 0.64j
US-HAD3	80	20	1.03 ± 0.02f	5.50 ± 0.02e	1.63 ± 0.25h	2.10 ± 0.48f
US-HAD4	60	40	1.57 ± 0.05b	8.46 ± 0.02b	2.44 ± 0.27b	2.86 ± 0.47b
US-HAD5	70	40	0.49 ± 0.00k	1.44 ± 0.01i	1.81 ± 0.16f	0.87 ± 0.39l
US-HAD6	80	40	0.74 ± 0.01h	1.41 ± 0.01j	1.44 ± 0.30j	1.84 ± 0.50i
US-HAD7	60	60	0.52 ± 0.01j	5.45 ± 0.02f	1.95 ± 0.19d	2.33 ± 0.64e
US-HAD8	70	60	0.68 ± 0.05i	7.26 ± 0.03c	1.54 ± 0.29i	2.48 ± 0.09d
US-HAD9	80	60	0.38 ± 0.01l	1.00 ± 0.00l	1.17 ± 0.28l	2.62 ± 0.17c
**IRD drying**						
CR1	60	–	0.79 ± 0.00h	1.62 ± 0.03k	1.93 ± 0.17i	2.80 ± 0.46i
CR2	70	–	0.66 ± 0.06i	1.65 ± 0.04j	2.54 ± 0.24c	2.93 ± 0.59d
CR3	80	–	1.22 ± 0.05f	4.85 ± 0.23e	1.99 ± 0.23h	2.86 ± 0.64e
US-IR1	60	20	3.24 ± 0.01a	7.396 ± 0.15a	2.76 ± 0.33a	3.30 ± 0.43a
US-IR2	70	20	0.56 ± 0.00j	2.06 ± 0.08i	1.67 ± 0.23k	2.60 ± 0.45k
US-IR3	80	20	1.95 ± 0.12e	2.71 ± 0.28g	2.28 ± 0.24e	1.96 ± 0.46l
US-IR4	60	40	2.69 ± 0.08b	5.25 ± 0.22b	2.67 ± 0.29b	3.18 ± 0.40b
US-IR5	70	40	0.85 ± 0.06g	4.91 ± 0.13c	2.20 ± 0.18g	2.79 ± 0.40j
US-IR6	80	40	2.42 ± 0.07c	4.86 ± 0.24d	1.80 ± 0.20j	2.81 ± 0.47g
US-IR7	60	60	2.41 ± 0.08d	2.61 ± 0.23h	2.51 ± 0.25d	2.86 ± 0.46f
US-IR8	70	60	0.50 ± 0.01k	3.28 ± 0.11f	2.28 ± 0.32f	3.12 ± 0.43c
US-IR9	80	60	0.38 ± 0.01l	1.50 ± 0.02l	1.32 ± 0.04l	2.81 ± 0.25h
**Temperature (T)**			<0.0001***	<0.0001***	<0.0001***	<0.0001***
**Frequency (F)**			<0.0001***	<0.0001***	<0.0001***	<0.0001***
**T × F**			<0.0001***	<0.0001***	<0.0001***	<0.0001***

Means followed by different letters within columns are significantly different (n.s., *, ***, nonsignificant, or significant at p < 0.05, or p < 0.001, respectively).

The US-treated samples had the most significant antioxidant activities in terms of the ABTS, DPPH, FRAP, and reducing power analysis, as shown in Table 5. When the antioxidant capacities of the four different assays were compared, it was discovered that the pretreated sweet potato samples followed a pattern: US-HAD1 > US-HAD4 > US-IR1 > US-IR4. The samples' higher antioxidant activity (ABTS, DPPH, FRAP, and reducing power assays) might be attributable to the US-treated sample’s shorter drying time than the control (as indicated previously), as the shorter drying period holds on a higher level of the antioxidant characteristics. These findings corroborate with An et al. [42], who described that food products must be dried for a shorter period and at a lower temperature (60 °C) to retain their antioxidant properties. According to Yilmaz et al. [52], numerous agricultural products that undergo the Maillard reaction after drying generally exhibit potent antioxidant activity due to a chain-breaking mechanism [53]. Our findings suggest enhanced antioxidant activities are often attributed to elevated TPC levels. As a result, the antioxidant content of the pretreated dried sweet potato samples is comparable to the TPC results. Ren et al. [6] demonstrated that the higher antioxidant activity observed may improve antioxidant element extraction efficiency after drying. Wang et al. [38] found that US pretreatment improved antioxidant capacity in dried cherry tomatoes. Udomkun et al.[54] discovered that samples with intense browning had significantly higher antioxidant capacity than untreated samples after osmotic dehydration pretreatment of papaya.

### 3.5 Enzyme inactivation analysis

#### 3.5.1 Polyphenol Oxidase (PPO)

The PPO activity of dried samples was greater than that of fresh ones (Table 6). Generally, PPO activity is increased when the membrane-bound enzymes are tended to release. A similar effect was noted during the high-pressure processing of strawberries [55]. Regarding the impact of drying process factors on PPO activity, neither drying temperature nor air velocity demonstrated a significant trend. However, when the US frequency was enhanced, the PPO activity in sweet potatoes was reduced.

**Table 6:** Effect of US pretreatments with HAD and IRD drying methods on enzyme inactivation of sweet potatoes.

Sample codes	Temperature ^o^C	Frequency kHz	PPO %	POD %	Enzymatic (%)	Non-enzymatic (%)
**HAD**						
Fresh	–	–	23.04 ± 0.54	16.23 ± 0.98		
CTL1	60	–	137.53 ± 0.14c	99.18 ± 0.35g	0.32 ± 0.12k	0.35 ± 0.007k
CTL2	70	–	135.19 ± 0.15d	97.67 ± 0.54h	0.41 ± 0.13i	0.43 ± 0.13c
CTL3	80	–	105.60 ± 0.03g	117.57 ± 0.12b	0.62 ± 0.006d	0.37 ± 0.003h
US-HAD1	60	20	85.49 ± 0.14j	77.58 ± 0.19l	0.69 ± 0.001a	0.40 ± 0.15e
US-HAD2	70	20	102.78 ± 0.13h	102.92 ± 0.30f	0.33 ± 0.15j	0.39 ± 0.006f
US-HAD3	80	20	138.31 ± 0.02b	106.63 ± 0.16d	0.61 ± 0.008e	0.46 ± 0.14a
US-HAD4	60	40	107.35 ± 0.02f	92.72 ± 0.36j	0.67 ± 0.003b	0.42 ± 0.16d
US-HAD5	70	40	234.75 ± 0.14a	118.67 ± 0.35a	0.55 ± 0.14f	0.31 ± 0.14l
US-HAD6	80	40	107.88 ± 0.01e	86.63 ± 0.09k	0.54 ± 0.02g	0.43 ± 0.13b
US-HAD7	60	60	95.93 ± 0.02i	106.94 ± 0.03c	0.63 ± 0.003c	0.36 ± 0.002j
US-HAD8	70	60	49.02 ± 0.15l	96.91 ± 0.24i	0.27 ± 0.10l	0.37 ± 0.00i
US-HAD9	80	60	71.86 ± 0.01k	104.20.29e	0.44 ± 0.14h	0.38 ± 0.002g
**IRD drying**						
CR1	60	–	10.53 ± 0.15k	66.78 ± 0.13g	3.39 ± 0.10f	2.50 ± 0.14f
CR2	70	–	22.04 ± 0.05e	68.82 ± 0.16f	2.11 ± 0.14j	1.37 ± 0.05j
CR3	80	–	12.87 ± 0.14h	14.14 ± 0.15j	4.06 ± 0.14c	3.09 ± 0.22e
US-IR1	60	20	14.93 ± 0.13f	17.76 ± 0.04i	4.38 ± 0.12a	0.79 ± 0.25l
US-IR2	70	20	35.34 ± 0.14b	101.92 ± 0.15c	1.41 ± 0.19k	1.85 ± 0.20g
US-IR3	80	20	11.14 ± 0.12j	12.64 ± 0.15k	3.87 ± 0.13e	4.14 ± 0.55a
US-IR4	60	40	24.02 ± 0.12d	107.32 ± 0.42b	4.25 ± 0.07b	1.16 ± 0.13k
US-IR5	70	40	46.35 ± 0.12a	109.70 ± 0.15a	1.33 ± 0.16l	3.28 ± 0.16d
US-IR6	80	40	13.36 ± 0.13g	25.16 ± 0.13h	4.00 ± 0.19d	3.35 ± 0.25b
US-IR7	60	60	26.10 ± 0.18c	85.68 ± 0.17e	2.60 ± 0.10i	1.59 ± 0.23i
US-IR8	70	60	11.55 ± 0.12i	93.09 ± 0.19d	3.27 ± 0.25h	1.69 ± 0.32h
US-IR9	80	60	10.52 ± 0.16l	10.34 ± 0.15l	3.35 ± 0.09g	3.29 ± 0.18c
**Temperature (T)**			<0.0001***	<0.0001***	<0.0001***	<0.0001***
**Frequency (F)**			<0.0001***	<0.0001***	<0.0001***	<0.0001***
**T × F**			<0.0001***	<0.0001***	<0.0001***	<0.0001***

Means followed by different letters within columns are significantly different (n.s., *, ***, nonsignificant, or significant at p < 0.05, or p < 0.001, respectively).

The procedure carried out at a US frequency of 60 kHz in both dryers at 80 °C resulted in the greatest drop-in PPO activity. With ultrasound treatment, more significant PPO activity was found at 70 °C in US-HAD5 and US-IR5 at 70 °C. Ultrasound of golden apple juice at 40 and 60 °C had a similar effect on PPO activity but a lower rate of inactivation (20 %) [56]. The disparity implies that the PPO of each apple type is susceptible to ultrasound treatment in various ways. In the high-pressure processing of plums and strawberries, a similar amount of inactivation was reported [55]. Because of the similarities in activation levels, the acoustic sonic technique is an appealing choice for PPO inactivation because it is less expensive than the high-pressure process. The activity of PPO in dried sweet potatoes showed a significant variation, supporting the impact of the acoustic treatment. When ultrasound was used, temperature changes did not affect PPO activity. Thermal processing, however, showed that as the temperature increased, the enzyme's activity dropped off significantly.

#### 3.5.2 Peroxidase (POD)

Compared to control and fresh samples, sweet potato POD activity increased following ultrasound pretreatment (Table 6). An increase in POD activity could result from hydrogen peroxide (H2O2) generation via ultrasonic treatment since this enzyme is triggered by the rise in H_2_O_2_ degrees, which is the enzyme's primary substrate. The present findings contrast with Jang & Moon [57], who discovered a slight decrease in the Peroxidase activity of Fuji apple samples treated with 40 kHz ultrasound. This disparity could be attributed to the different types of plant species compared to the current study and the different ultrasonic frequencies used in their research. PPOs are highly resistant to heat processing, significantly below 80 °C [58]. The results reported herein verify this and suggest that the energy delivered to the samples by ultrasonic treatment may decrease the endurable temperature of PODs.

### 3.6 Enzymatic and non-enzymatic browning

According to Table 6, there was a significant increase in enzymatic browning value at lower US-frequency (20 kHz), where a significantly (*p < 0.05*) high enzymatic browning value was identified in hot-air (US-HAD1, 0.69) and infrared (US-IR1, 4.38) drying. The decrease in enzymatic browning in samples was noted with the increase in US frequencies and temperature in both dryers. This loss may be explained by the extended drying period at high temperatures (70 and 80 °C) and the enzymatic browning response throughout the sweet potato drying process since temperature and drying time are essential variables in color degradation. In all US-pretreated samples, a comparable effect of temperature on the lowering of enzymatic levels was seen. POD and PPO enzymes cause enzymatic browning pigmentation as they affiliate with endogenic phenolic chemicals while drying the fruit samples [54].

Nevertheless, the contradictory effect of temperature and US frequency on the maintenance of non-enzymatic browning during the drying process was observed. Higher temperatures with lower US frequencies were seen to retain the non-enzymatic value compared to control samples, i.e., US-HAD (0.46 %) and US-IR3 (4.14 %) obtained the highest values at 20 kHz and 80 °C. This could cause the drying chamber's higher moisture level, diluting the released reactant species and delaying the chemical activities in damaged cells. Higher non-enzymatic browning values are most likely caused by brown polymers produced by the Maillard reaction. They rise at high internal temperatures of drying materials due to a drop in US frequencies. Udomkun et al. [54] proposed that phenolic acid and Vit-C oxidation might form complex brown pigments. Again, lower US frequencies encouraged non-enzymatic coloration as high values were obtained. This could be because the ultrasonic waves' massive disruption of cell integrity caused more reactant species to be released, resulting in further browning.

#### 3.6.1 Browning index (BI)

The browning index of sweet potatoes was affected by various pretreatments and drying procedures (Table 4). Compared to HAD, IRD had a higher browning index. This could be associated with the Maillard reaction and the oxidation of Vit-C. In both dryers, ultrasound significantly reduced the BI values compared to the controls. Ultrasound pretreatment samples with lower BI values show that ultrasound slowed down BI production. Cernîşev [59] found the BI rate rose from 0.585 to 0.684 when dried tomato quarters were heated to various temperatures.

### 3.7 Surface color

The influence of temperature on the total color difference (ΔE) in US pretreated, HAD, and IRD dried samples is tabulated in Supplementary Table-S4. The 20 and 60 kHz ultrasound frequencies in the US-treated samples were significantly different in ΔE in US-IR1 at 80 °C (30.28), followed by US-IR7 at 60 °C (29.76). Compared to US pretreated samples dried in HAD, the IR drying technique with US pretreatments showed the higher ΔE values. Even though the drying period was shorter in IRD drying (Fig. 2), the drying temperature was high enough to intensify the non-enzymatic browning reaction, resulting in color alterations. When IR drying with US pretreatment was compared to HAD drying, the speed and intensity of ΔE were substantially faster. Due to the uneven nature of IR heating, the samples may burn during the procedure, and subsequently, color variation was examined more thoroughly than in the other samples. Rashid et al. [9] investigated the effect of US and IRD heating on sweet potato drying. They found that higher IR heating instigates high internal pressure, promoting color changes in the sample. Supplementary Table-S4 shows that a rise in the drying temperature induces higher ΔE values in all pretreated ultrasonic samples in HAD drying. Chemical reactions involving colorants, e.g., the Millard reaction and the synthesis of melanoidins during the drying, could explain the variation in the ΔE at various temperature ranges. These results are comparable with Bromberger et al. [60]. The rise in US frequencies (20–60 kHz) and IRD temperature range lead to a decline in ΔE. However, lower temperature influences the nutrients characteristics by lengthening the drying time, increasing ΔE. As a result, ultrasound does not affect ΔE by leaving chemical reactions unaltered; instead, it has a more physical function, and Cell wall disintegration happens in the samples' interior tissue. These findings are consistent with Bromberger et al. [60] and Shahidi et al. [10]. According to a previous study, dried products with a lower ΔE value are of higher quality [14]. Thus, despite being non-destructive, ultrasonic waves damaged the cell walls of tissues and caused the samples to dry faster when exposed to different temperature ranges. As a result, in various experimental treatments, the ΔE value was reduced, demonstrating the improved quality of the samples. According to statistical analysis (T × F interaction), significant differences (*p < 0.05*) have been found (Supplementary Table-S4). The average hue value (h°) of the fresh, US-HAD, and US-IR samples was 79.09°, 69.25–77.62^o,^ and 79.32–76.47°, respectively. h° values of US-HAD (dried at 60,70 and 80 °C) and IRD samples were found significantly (*p < 0.05*) lower (or nearly similar in range) than fresh and control samples dried in HAD. According to Wiktor et al. [7], the h° values remained unchanged or higher for ultrasonic pretreated (by immersion) dried apple samples compared to the untreated sample. Kahraman et al. [61] reported that hue angle was significantly within different drying methods of apple slices, which supports our findings. The quantitative feature of colorfulness, known as chroma (C*), measures the degree of hue difference compared to a grey color of the same light. The results showed that the changes in C* were greater between the two drying methods for orange sweet potatoes. The samples treated in US-HAD and US-IRD were significantly lower than fresh samples. The US pretreated samples lost soluble solids (including color pigments), accounting for the lower chroma values. C* values were higher in the samples subjected to two drying techniques. The samples treated with US-HAD and US-IRD were much lower in C* values than fresh samples (Supplementary Table-S4) because US pretreated samples lost soluble solids (including color pigments).

Although the US pretreated and control samples were found non-significant, however, the C* values of the US pretreated samples were higher in both drying techniques than the control samples, which might be related to the influence of ultrasound cavitation, revealing the yellow pigment attached to the intracellular structure of the samples. Moreover, higher water loss leads to solid gain, resulting in color saturation. Costa et al. [62] demonstrated that the sonicated pineapple juice had a higher C* value than non-sonicated juice samples. samples (*p > 0.05*). Samples from both dryers significantly differed from the fresh samples (*p > 0.05*). Since ultrasonic pretreatment significantly improved the yellow appearance of the sweet potato, identifying the presence of β-carotene [63]. Except for a few, significant alteration in the whiteness index (WI) was observed amongst the samples. The WI of the US-HAD and US-IRD samples varied significantly (*p < 0.05*) compared with control and fresh samples. The higher WI values were noted in US-HAD5 (70.50) and US-IR5 (68.44) at 70oC and 40 kHz US frequency. Compared to the US-treated, WI values were lower in control samples, that might be related to the inactivation of peroxidase and polyphenol oxidase, which causes browning and thus preserves sample WI [64].

### 3.8 Texture profile analysis (TPA)

The hardness and resilience of US-pretreated samples did not vary significantly (*p > 0.05*) compared to untreated samples in hot-air and infrared drying (Table 7). According to Santacatalina et al. [65], increasing the amount of applied ultrasonic power led to a low but non-significant (p < 0.05) decline in sample hardness. Brncic et al. [66] found that increasing the US intensity from 50 % to 100 % amplitude increased the dried apple’s hardness. Zhang et al. [67] discovered the same phenomenon with dried strawberry chips treated with the US having significantly higher hardness than control samples. Song et al. [68] linked the elevated hardness levels of the US pretreated samples with declined moisture content. The higher resilience in the matrix may account for the increased resilience in the US-pretreated samples. The findings revealed that sweet potato slices subjected to HAD drying, including control and US pretreated samples, had better textural qualities than infrared drying.

**Table 7:** Effect of US pretreatments with HAD and IR drying methods on sweet potato textural profile analysis (TPA).

Sample codes	Hot-air drying		Infrared drying	
	Hardness	Resilience	Hardness	Resilience
Control1	98.8c	24.75c	224.1b	15.31c
Control2	107.5c	30.15bc	192.7b	24.91abc
Control3	111.4c	32.3abc	319.8ab	22.51bc
USPS1	238bc	42.78ab	519.8ab	43.27ab
USPS2	256bc	36.28abc	494ab	45.76ab
USPS3	354bc	36.97abc	618ab	37.6abc
USPS4	144.1c	35.41abc	511ab	47.67a
USPS5	265bc	36.23abc	524.9ab	35.96abc
USPS6	424bc	43.81a	362.1ab	33.13abc
USPS7	542.3abc	37.86ab	928.8a	34.75abc
USPS8	872.2a	38.21ab	912.8a	35.12abc
USPS9	728abc	40.92ab	640ab	41.79ab

USPS = Ultrasound pretreated samples.

Means followed by different letters within columns are significantly different (n.s., *, ***, nonsignificant, or significant at p < 0.05, or p < 0.001, respectively).

## 4 Conclusion

The current study comprised an innovative approach of drying sweet potatoes using ultrasound pretreatments with two drying methods since a synergistic application of these methods with a comprehensive set of experiments and fifteen predicting kinetic models were not conducted so far. Control samples dried slower and at a higher rate than ultrasound-treated samples in both HAD and IR methods. The Hii model was the most accurate for predicting sweet potato drying for HAD and IRD drying. US frequencies, such as 20 and 40 kHz (US-HAD/IR 1–6) at 60 and 70 °C, were found to be appropriate, whereas 60 kHz (US-HAD/IR 7–9) US frequency (at 80 °C) resulted in poor quality attributes in terms of preservation and high hardness of the samples where specific pretreatment settings were insufficient. This study indicates that 20 min is a critical duration during which the drying rate in all ultrasonic treatments tends to decrease when compared to control samples. Sweet potato slices at 40 kHz US frequency in HAD demonstrated improved textural properties than IRD dried samples. Finally, this study showed that ultrasonic pretreatments combined with hot-air and infrared drying could result in an energy-efficient method with high-quality retention in dried sweet potatoes.

### CRediT authorship contribution statement

**Muhammad Tayyab Rashid:** Data curation, Methodology, Writing – original draft. **Kunlun Liu:** Conceptualization, Supervision. **Mushtaque Ahmed Jatoi:** Writing – review & editing. **Bushra Safdar:** Writing – review & editing. **Dingyang Lv:** Visualization. **Dengzhong Wei:** Software.

## Declaration of Competing Interest

The authors declare that they have no known competing financial interests or personal relationships that could have appeared to influence the work reported in this paper.
