# Supplementary material for: Developing ultrasound-assisted hot-air and infrared drying technology for sweet potatoes
Source: Ultrason Sonochem. 2022 May 20;86:106047. doi: 10.1016/j.ultsonch.2022.106047 (PMC9136187; doi:10.1016/j.ultsonch.2022.106047)
Supplement: Supplementary data 1 [file mmc1.docx]

**SUPPLEMENTARY MATERIAL**

**Supplementary Table S1:** Applied mathematical models for drying sweet potatoes using US pretreatments with HAD and IR drying methods.

| **S. No** | **Model name** | **Model** | **References** |
| --- | --- | --- | --- |
| 1 | Newton | $Mr=exp(-kt)$ | [1] |
| 2 | Page | $Mr=exp(-kt^{n}$) | [2] |
| 3 | Henderson and Pabis | $Mr=aexp(-kt^{n}$) | [3] |
| 4 | Modified Henderson and Pabis | $Mr=aexp\left( -kt \right)+bexp\left( -gt \right)+cexp(-ht)$ | [4] |
| 5 | Logarithmic model | $Mr=aexp\left( -kt \right)+c$ | [5] |
| 6 | Midilli model | $Mr=aexp\left( -kt \right)+bt$ | [6] |
| 7 | Two-term model | $Mr=aexp\left( {-k}_{1}t \right)+bexp\left( {-k}_{2}t \right)$ | [7] |
| 8 | Hii model | $Mr=aexp\left( {-k}_{1}t^{n} \right)+bexp\left( {-k}_{2}t^{n} \right)$ | [8] |
| 9 | Verma Model | $Mr=aexp\left( -kt \right)+\left( 1-a \right)exp(-gt)$ | [9] |
| 10 | Modified Midilli model | $Mr=aexp\left( -kt \right)+b$ | [10] |
| 11 | Aghbashlo model | $Mr=exp\left( k_{1}t/1+k_{2}t \right)$ | [11] |
| 12 | Wang and Singh | $Mr=1+at+bt^{2}$ | [12] |
| 13 | Silva's model | $Mr=exp(-at-b\sqrt{t})$ | [13] |

**Supplementary Table S2.** Averages of selected models fitted to thin-layer drying using the US and HAD for sweet potatoes

| **Sample Codes** | **T**  **(^°^C)** | **Coefficients** | **R^2^** | **RSS** | **χ^2^** | **RMSE** |
| --- | --- | --- | --- | --- | --- | --- |
| **Modified Henderson and Pabis model** | | | | | | |
| CTL1  CTL2  CTL3  US-HAD1  US-HAD2  US-HAD3  US-HAD4  US-HAD5  US-HAD6  US-HAD7  US-HAD8  US-HAD9 | 60  70  80  60  70  80  60  70  80  60  70  80 | a.1.952, b.0-.472, k.0.011, g. c.0.009, -0.472, h. 0.009  a.0.730, b.0.142, k.0.016, g.0.016, c.0.142, h. 0.016  a.57.381, b.42.25, k.0.007, g.0.005, c.-0.98.62, h. 0.006  a.0.777, b. 0.436, k.0.019 g.0.007, c.-0.216, h.0.007  a.1.26, b.0.859, k.0.013, g.0.013, c.-1.12, h.0.012  a.1.24, b.0.758, k.0.016, g.0.016, c.-0.996, h.0.014  a.0.134, b.0.434, k.0.045, g.0.015, c.0.434, h.0.015  a.67.81, b.55.65, k.0.009, g.0.008, c.-122.46, h.0.008  a.1.36, b.0.916, k.0.016, g.0.016, c.-1.26, h..012  a.0.940, b.0.033, k.0.015, g.0.001, c.0.032, h.0.001  a.28.49, b.21.75, k.0.007, g.0.006, c.-49.23, h.0.006  a.0.703, b.0.157, k.0.015, g.0.015, c.0.157, h.0.015 | 0.999  0.998  0.999  0.998  1.000  0.998  0.999  0.999  0.995  1.000  0.997  0.996 | 0.00  0.00  0.00  0.00  0.00  0.00  0.00  0.00  0.00  0.00  0.00  0.00 | 6.90×10^-5^  0.00017  0.00018  0.00018  9.40×10^-5^  7.30×10^-5^  0.00018  0.00067  0.00067  5.30×10^-5^  0.00025  0.00049 | 0.0076  0.0001  0.0001  0.0001  0.0003  0.0001  0.0001  0.0001  0.0006  0.0064  0.0002  0.0004 |
| **Henderson and Pabis model** | | | | | | |
| CTL1  CTL2  CTL3  US-HAD1  US-HAD2  US-HAD3  US-HAD4  US-HAD5  US-HAD6  US-HAD7  US-HAD8  US-HAD9 | 60  70  80  60  70  80  60  70  80  60  70  80 | k.0.938, n.0.15, a.0562  k.0.805, n.0.46, a.0.563  k.0.787, n.0.70, a.0.560  k.0.823, n.0.21, a.0.545  k.0.769, n.0.16, a.0.571  k.0.766, n.0.08, a.0.545  k.0.456, n.0.69, a.0.512  k.0.364, n.0.39, a.0.483  k.0.684, n.0.52, a.0.518  k.0.279, n.0.82, a.0.527  k.0.627, n.0.98, a.0.508  k.0.824, n.0.34, a.0.579 | 0.439  0.529  0.308  0.488  0.500  0.698  0.279  0.519  0.396  0.350  0.483  0.308 | 0.54  0.43  0.23  0.68  0.12  0.34  0.50  0.49  0.32  0.36  0.21  0.67 | 0.06545  0.03544  0.06843  0.03545  0.03549  0.03166  0.09848  0.06483  0.03219  0.03244  0.03540  0.03214 | 0.3216  0.6456  0.3546  0.5433  0.3540  0.1230  0.1873  0.3546  0.3214  0.6873  0.2146  0.3547 |
| **Logarithmic model** | | | | | | |
| CTL1  CTL2  CTL3  US-HAD1  US-HAD2  US-HAD3  US-HAD4  US-HAD5  US-HAD6  US-HAD7  US-HAD8  US-HAD9 | 60  70  80  60  70  80  60  70  80  60  70  80 | a.1.031, k.0.013, c.-0.022  a.1.029, k.0.015, c.-0.021  a.0.995, k.0.019, c. 0.017  a.0.951, k.0.016, c. 0.043  a.1.012, k.0.015, c.-0.009  a.1.022, k.0.019, c. -0.013  a.0.974, k.0.017, c.0.020  a.0.980, k.0.019, c.0.030  a.1.066, k.0.021, c.-0.054  a.0.952, k.0.015, c.0.053  a.0.969, k.0.013, c.0.040  a.1.067, k.0.013, c-0.065 | 0.999  0.998  0.998  0.998  0.999  0.998  0.998  0.999  0.995  1.000  0.997  0.998 | 0.00  0.00  0.00  0.00  0.00  0.00  0.00  0.00  0.00  0.00  0.00  0.00 | 9.60×10^-5^  0.00014  0.00023  0.00017  0.00011  9.30×10^-5^  0.00017  0.00001  0.00066  5.30×10^-5^  0.00027  0.00019 | 0.0090  0.0111  0.01230.0129  0.0015  0.0654  0.0634  0.0022  0.0035  0.0087  0.0001  0.0035 |
| **Midilli and others model** | | | | | | |
| CTL1  CTL2  CTL3  US-HAD1  US-HAD2  US-HAD3  US-HAD4  US-HAD5  US-HAD6  US-HAD7  US-HAD8  US-HAD9 | 60  70  80  60  70  80  60  70  80  60  70  80 | a.1.01, b.0.27, k.0.014  a.1.009, b.0.05, k.0.016  a.1.013, b.0.000, k.0.019  a.0.992, b.0.000, k.0.015  a.0.100, b.0.720, k.0.015  a.1.010, b.0.759, k.0.019  a.0.993, b.0.334, k.0.017  a.1.010, b.0.000, k.0.019  a.1.013, b.0.000, k.0.022  a.1.001, b.0.000, k.0.014  a.1.008, b.0.000, k.0.013  a.1.004, b.0.000, k.0.014 | 0.998  0.998  0.998  0.998  0.999  0.998  0.998  0.999  0.994  0.999  0.997  0.998 | 0.00  0.00  0.00  0.00  0.00  0.00  0.00  0.00  0.00  0.00  0.00  0.00 | 9.60×10^-5^  0.00014  0.00023  0.00017  9.90×10^-5^  0.00012  0.00017  0.00067  0.00001  6.90×10^-5^  0.00027  0.00033 | 0.0090  0.0114  0.0123  0.0156  0.0025  0.0984  0.0315  0.0354  0.0165  0.0345  0.0314  0.0354 |
| **Modified Middili and others** | | | | | | |
| CTL1  CTL2  CTL3  US-HAD1  US-HAD2  US-HAD3  US-HAD4  US-HAD5  US-HAD6  US-HAD7  US-HAD8  US-HAD9 | 60  70  80  60  70  80  60  70  80  60  70  80 | a.1.031, k.0.013, b.-0.022  a.1.029, k.0.015, b.-0.021  a.0.0995, k.0.019, b.0.017  a.0.0951, k.0.16, b.0.043  a.1.012, k.0.015, b.-0.009  a.1.012, k.0.019, b.-0.013  a.0.974, k.0.017, b.0.020  a.0.980, k.0.019, b.0.030  a.1.066, k.0.021, b.-0.054  a.0.952, k.0.015, b.0.053  a.0.969, k.0.013, b.0.040  a.1.067, k.0.013, b.-0.065 | 0.999  0.998  0.998  0.998  0.999  0.998  0.998  0.999  0.995  1.000  0.997  0.998 | 0.00  0.00  0.00  0.00  0.00  0.00  0.00  0.00  0.00  0.00  0.00  0.00 | 9.60×10^-5^  0.00014  0.00023  0.00017  0.00011  9.05×10^-5^  0.00017  0.00035  0.00016  5.30×10^-5^  0.00027  0.00029 | 0.0090  0.0111  0.0123  0.0364  0.0328  0.0967  0.0349  0.0766  0.0321  0.0254  0.0540  0.0943 |
| **Verma and other models** | | | | | | |
| CTL1  CTL2  CTL3  US-HAD1  US-HAD2  US-HAD3  US-HAD4  US-HAD5  US-HAD6  US-HAD7  US-HAD8  US-HAD9 | 60  70  80  60  70  80  60  70  80  60  70  80 | a.-9.461, k.0.010, g.0.010  a.-11.917, k.0.011, g. 0.012  a.-0.363, k.0.018, g.0.018  a.0.300, k.0.008, g.0.020  a.-3.828, k.0.012, g.0.013  a.-9.379, k.0.015, g. 0.015  a.0.877, k.0.015, g.0.047  a.0.002, k.0.016, g.0.018  a.-12.852, k.0.014, g.0.014  a.0.054, k.0.000, g.0.015  a.-0.541, k.0.012, g.0.012  a.-7.761, k.0.009, g.0.009 | 0.999  0.999  0.998  0.998  1.000  0.998  0.999  0.999  0.995  1.000  0.996  0.998 | 0.00  0.00  0.00  0.00  0.00  0.00  0.00  0.00  0.00  0.00  0.00  0.00 | 9.00×10^-5^  0.00013  0.00023  0.00018  0.00011  7.30×10^-5^  0.00018  0.00017  0.00077  5.30×10^-5^  0.00038  0.00019 | 0.0091  0.0094  0.0123  0.0160  0.0143  0.0023  0.0126  0.0031  0.0032  0.0132  0.0654  0.0135 |
| **Silva and others model** | | | | | | |
| CTL1  CTL2  CTL3  US-HAD1  US-HAD2  US-HAD3  US-HAD4  US-HAD5  US-HAD6  US-HAD7  US-HAD8  US-HAD9 | 60  70  80  60  70  80  60  70  80  60  70  80 | a.0.016, b.0.016  a.0.018, b.0.017  a.0.019, b.-0.008  a.0.012, b.0.023  a.0.016, b.-0.006  a.0.022, b.-0.019  a.0.014, b.0.016  a.0.018, b.0.001  a.0.030, b.-0049  a.0.011, b.0.011  a.0.011, b.0.005  a.0.017, b.-0.021 | 0.999  0.999  0.998  0.998  1.000  0.999  0.998  0.998  0.997  0.997  0.996  0.997 | 0.00  0.00  0.00  0.00  0.00  0.00  0.00  0.00  0.00  0.00  0.00  0.00 | 6.20×10^-5^  0.00010  0.00017  0.00019  9.40×10^-5^  1.60×10^-5^  0.00012  0.00021  0.00065  0.00020  0.00040  0.00031 | 0.0072  0.0097  0.0108  0.0354  0.0651  0.0231  0.0684  0.0198  0.0126  0.0165  0.0014  0.0156 |
| **Aghbashlo and others mode** | | | | | | |
| CTL1  CTL2  CTL3  US-HAD1  US-HAD2  US-HAD3  US-HAD4  US-HAD5  US-HAD6  US-HAD7  US-HAD8  US-HAD9 | 60  70  80  60  70  80  60  70  80  60  70  80 | k1.6.065, k2.-6.079  k1.-2.35, k2.0.219  k1.-0.260, k2.0.242  k1.-0.153, k2.0.139  k1.-0.317, k2.0.0301  k1.-0.256, k2.0.237  k1.-3.410, k2.3.394  k1.-0.001, k2.-0.016  k1.-0.580, k2.0.557  k1.1.216, k2.-1.229  k1.-2.291, k2.2.279  k1.0.345, k2.-0.360 | 0.998  0.998  0.998  0.995  0.999  0.998  0.997  0.998  0.992  0.994  0.996  0.995 | 0.00  0.00  0.00  0.00  0.00  0.00  0.00  0.00  0.01  0.00  0.00  0.00 | 0.00020  0.00025  0.00023  0.00044  9.40×10^-5^  0.00032  0.00065  0.00041  0.00096  0.00019  0.00063  0.00075 | 0.0130  0.0150  0.01230.0136  0.0354  0.0332  0.0987  0.0350  0.0653  0.0231  0.0643  0.0247 |
| **Page Model** | | | | | | |
| CTL1  CTL2  CTL3  US-HAD1  US-HAD2  US-HAD3  US-HAD4  US-HAD5  US-HAD6  US-HAD7  US-HAD8  US-HAD9 | 60  70  80  60  70  80  60  70  80  60  70  80 | k.0.010, n.1.077  k.0.012, n.1.076  k.0.016, n.1.020  k.0.023, n.0.897  k.0.014, n.1.024  k.0.015, n.1.069  k.0.021, n.0.936  k.0.019, n.0.983  k.0.011, n.1.179  k.0.020, n.0.901  k.0.014, n.0.963  k.0.009, n.1.114 | 0.999  0.999  0.998  0.998  0.999  0.999  0.999  0.998  0.998  0.998  0.996  0.998 | 0.00  0.00  0.00  0.00  0.00  0.00  0.00  0.00  0.00  0.00  0.00  0.00 | 6.16×10^-5^  6.69×10^-5^  0.00016  0.00018  9.38×10^-5^  4.66×10^-5^  0.00018  0.00015  0.00036  0.00016  0.00063  0.00073 | 0.0072  0.0078  0.01060.0638  0.0267  0.0035  0.0135  0.0054  0.0654  0.0546  0.0354  0.0983 |
| **Wang and Singh Model** | | | | | |  |
| CTL1  CTL2  CTL3  US-HAD1  US-HAD2  US-HAD3  US-HAD4  US-HAD5  US-HAD6  US-HAD7  US-HAD8  US-HAD9 | 60  70  80  60  70  80  60  70  80  60  70  80 | a-0.010, b.0.59  a.-0.012, b.0.99  a.-0.014, b.0.18  a.-0.011, b.0.16  a.-0.012, b.0.79  a.-0.015, b.0.19  a.-0.012, b.0.70  a.-0.014, b.0.23  a.-0.018, b.0.18  a.-0.009, b.0.35  a.-0.010, b.0.60  a.-0.011, b.0.50 | 0.968  0.998  0.996  0.992  0.973  0.997  0.974  0.998  0.998  0.969  0.998  0.997 | 0.01  0.00  0.01  0.02  0.01  0.00  0.09  0.02  0.01  0.02  0.01  0.00 | 0.00068  0.00028  0.00090  0.00187  0.00085  8.70×10^-5^  0.00036  0.00065  0.00048  0.00226  0.00049  0.0002 | 0.0240  0.0160  0.0245  0.0637  0.0439  0.0852  0.0349  0.0761  0.0162  0.0863  0.0654  0.0345 |
| **Newton** | | | | | | |
| CTL1  CTL2  CTL3  US-HAD1  US-HAD2  US-HAD3  US-HAD4  US-HAD5  US-HAD6  US-HAD7  US-HAD8  US-HAD9 | 60  70  80  60  70  80  60  70  80  60  70  80 | k.0.014  k.0.016  k.0.018  k.0.015  k.0.015  k.0.019  k.0.016  k.0.018  k.0.023  k.0.013  k.0.012  k.0.015 | 0.998  0.998  0.998  0.995  0.999  0.998  0.997  0.998  0.992  0.995  0.996  0.995 | 0.00  0.00  0.00  0.00  0.00  0.05  0.01  0.00  0.00  0.00  0.01  0.00 | 0.00020  0.00025  0.00023  0.00043  9.43×10^-5^  0.00035  0.00065  0.00018  0.00085  0.00024  0.00037  0.00049 | 0.0130  0.0150  0.01230.0129  0.0169  0.0136  0.0167  0.0128  0.0137  0.0364  0.0837  0.0946 |

CTL= Control; US-HAD= Ultrasound hot air drying. CR= Control; US-IR= Ultrasound infrared drying

**Supplementary Table S3:** Averages of selected models fitted to thin-layer drying using the US and IR for sweet potatoes

| **Sample Codes** | **T**  **(^°^C)** | **Coefficients** | **R^2^** | **RSS** | **χ^2^** | **RMSE** |
| --- | --- | --- | --- | --- | --- | --- |
| **Modified Henderson and Pabis model** | | | | | | |
| CR1  CR2  CR3  US-HAD1  US-HAD2  US-HAD3  US-HAD4  US-HAD5  US-HAD6  US-HAD7  US-HAD8  US-HAD9 | 60  70  80  60  70  80  60  70  80  60  70  80 | k.0.005, b.129.79, a.-231.22, g.0.006, c.102.48, h.0.004  k.0.006, b.68.268, a.26.071, g.0.010, c.-93.33, h.0.009  k.0.020, b.1.882, a.-0.63, g.0.029, c.-0.392, h.0.020  k.-0.005, b.123.81, a.-208.18, g.-0.004, c.8538, h.-0.06  k.0.016, b.0.080, a.-20.35, g.1.438, c.21.27, h.0.016  k.0.035, b.0.705, a.0.186, g.0.035, c.0.186, h.0.035  k.-0.001, b.63.75, a.-106.27, g.0.00, c.43.53, h.-0.003  k.-0.054, b.1.002, a.0.910, g.0.026, c.-0.917, h.-0.054  k.-0.052, b.0.001, a.0.815, g.0.089, c.0.826, h.-0.089  k.0.026, b.0.086, a.0.912, g.1.101, c.0.002, h.-0.018  k.0.017, b.2.179, a.-0.631, g.0.025, c.-0.523, h.0.017  k.0.026, b.3.156, a.0.015, g.0.034, c.-0.918. h.0.035 | 0.984  0.998  0.990  0.990  0.999  0.962  0.999  0.999  0.864  0.996  0.999  0.992 | 0.02  0.00  0.01  0.01  0.00  0.02  0.00  0.00  0.00  0.00  0.00  0.00 | 0.00197  0.00018  0.00124  0.00101  6.80×10^-5^  0.00026  0.00018  6.90×10^-5^  0.00063  0.00029  0.00011  0.00078 | 0.0037  0.0056  0.0036  0.0082  0.0003  0.0019  0.0638  0.0007  0.0658  0.0259  0.0098  0.0161 |
| **Henderson and Pabis model** | | | | | | |
| CR1  CR2  CR3  US-HAD1  US-HAD2  US-HAD3  US-HAD4  US-HAD5  US-HAD6  US-HAD7  US-HAD8  US-HAD9 | 60  70  80  60  70  80  60  70  80  60  70  80 | k.0.903, n.-2.108, a.0.659  k.0.815, n.-1.408, a.0.551  k.0.782, n.-2.163, a.0.608  k.0.635, n.-2.153, a.0.348  k.0.519, n.-2.459, a.0.849  k.0.537, n.-2.079, a.0.437  k.0.591, n.-2.816, a.0.492  k.0.539, n.-2.917, a.0.534  k.0.519, n.-2.146, a.0.507  k.0.508, n.-2.349, a.0.392  k.0.534, n.-2.492, a.0.537  k.0.561, n.-2.937, a.0.592 | 0.637  0.349  0.538  0.369  0.348  0.439  0.673  0.295  0.348  0.438  0.308  0.208 | 0.73  0.41  0.35  0.63  0.15  0.96  0.34  0.96  0.15  0.68  0.96  0.37 | 0.06611  0.04566  0.05731  0.03649  0.03675  0.03686  0.03648  0.06423  0.06313  0.01564  0.06786  0.05676 | 0.2365  0.2045  0.2034  0.2364  0.2649  0.2364  0.2963  0.2459  0.2648  0.2694  0.2648  0.2964 |
| **Logarithmic model** | | | | | | |
| CR1  CR2  CR3  US-HAD1  US-HAD2  US-HAD3  US-HAD4  US-HAD5  US-HAD6  US-HAD7  US-HAD8  US-HAD9 | 60  70  80  60  70  80  60  70  80  60  70  80 | a.1.092, k.0.024, c.-0.022  a.0.996, k.0.034, c.0.015  a.1.097, k.0.036, c.-0.069  a.1.158, k.0.020, c.-0.109  a.1.063, k.0.030, c.-0.074  a.1.550, k.0.018, c.-0.510  a.1.146, k.0.021, c.-0.122  a.1.041, k.0.026, c.-0.044  a.1.666, k.0.004, c.-0.103  a.0.998, k.0.026, c.0.019  a.0.940, k.0.031, c.0.043  a.1.137, k.0.032, c.-0.11 | 0.981  0.996  0.989  0.978  0.999  0.998  0.988  0.999  0.995  0.996  0.997  0.991 | 0.02  0.00  0.01  0.03  0.00  0.01  0.00  0.00  0.00  0.00  0.01  0.00 | 0.00218  0.00035  0.00136  0.00239  0.00013  0.00072  0.00018  0.00364  0.00968  0.00367  0.00962  0.00364 | 0.0072  0.0015  0.0354  0.0654  0.0324  0.0212  0.0234  0.0354  0.0525  0.0564  0.0215  0.0345 |
| **Midilli and others model** | | | | | | |
| CR1  CR2  CR3  US-HAD1  US-HAD2  US-HAD3  US-HAD4  US-HAD5  US-HAD6  US-HAD7  US-HAD8  US-HAD9 | 60  70  80  60  70  80  60  70  80  60  70  80 | k.0.024, b.0.000, a.1.071  k.0.034, b.0.000, a.1.012  k.0.038, b.-0.001, a.1.030  k.0.021, b.-0.001, a.1.051  k.0.032, b.-0.001, a.0.990  k.0.023, b.-0.004, a.1.040  k.0.023, b.-0.001, a.1.026  k.0.026, b.0.000, a.0.998  k.0.043, b.-0.001, a.1.009  k.0.026, b.0.000, a.1.017  k.0.029, b.0.000, a.0.981  k.0.035, b.-0.001, a.1.027 | 0.981  0.996  0.989  0.977  0.998  0.987  0.995  0.999  0.994  0.996  0.997  0.991 | 0.00  0.00  0.00  0.00  0.00  0.00  0.09  0.15  0.10  0.00  0.00  0.00 | 0.00173  0.00034  0.00121  0.00063  0.00625  0.00036  0.00035  0.00369  0.00359  0.00364  0.00096  0.00056 | 0.0383  0.0176  0.03210.0167  0.0364  0.0638  0.0961  0.0628  0.0468  0.0967  0.0131  0.0101 |
| **Modified Middili and others** | | | | | | |
| CR1  CR2  CR3  US-HAD1  US-HAD2  US-HAD3  US-HAD4  US-HAD5  US-HAD6  US-HAD7  US-HAD8  US-HAD9 | 60  70  80  60  70  80  60  70  80  60  70  80 | a.1.092, k.0.024, b.-0.022  a.0.996, k.0.034, b.0.015  a.1.097, k.0.036, b.-0.069  a.1.158, k.0.020, b.-0.109  a.1.036, k.0.030, b.-0.74  a.1.550, k.0.018, b.-0.510  a.1.146, k.0.021, b.-0.122  a.1.041, k.0.026, b.-0.044  a.1.111, k.0.040, b.-0.103  a.0.998, k.0.026, b.0.019  a.0.940, k.0.031, b.0.043  a.1.137, k.0.032, b.-0.111 | 0.981  0.996  0.989  0.978  0.998  0.998  0.996  0.999  0.995  0.996  0.997  0.991 | 0.02  0.00  0.02  0.02  0.00  0.01  0.00  0.00  0.00  0.00  0.00  0.01 | 6.90×10^-5^  9.90×10^-5^  0.00023  0.00018  9.40×10^-5^  7.30×10^-5^  0.00018  0.00067  0.00067  5.30×10^-5^  0.00028  0.00019 | 0.0076  0.0094  0.0122  0.0129  0.0081  0.0100  0.0122  0.0257  0.0195  0.0064  0.0152  0.0107 |
| **Verma and other models** | | | | | |  |
| CR1  CR2  CR3  US-HAD1  US-HAD2  US-HAD3  US-HAD4  US-HAD5  US-HAD6  US-HAD7  US-HAD8  US-HAD9 | 60  70  80  60  70  80  60  70  80  60  70  80 | a.-16.73, k.0.014, g.0.015  a.-1.093, k.0.031 g.0.032  a.-14.78, k.0.023, g.0.023  a.-15.55, k.0.011, g.0.011  a.0.786, k.0.036, g.0.036  a.-13.664, k.0.008, g.0.009  a.-10.82, k.0.012, g.0.013  a.-2.666, k.0.019, g.0.021  a.-.754, k.0.025, g.0.027  a.-0.614, k.0.024, g.0.025  a.0.905, k.0.025, g.2.113  a.-13.259, k.0.019, g.0.020 | 0.976  0.996  0.989  0.976  0.996  0.985  0.995  0.999  0.995  0.996  0.999  0.991 | 0.03  0.00  0.01  0.03  0.00  0.01  0.01  0.00  0.00  0.00  0.01  0.01 | 0.00261  0.00037  0.00144  0.00368  0.00363  0.00560  0.00961  0.00129  0.00967  0.00648  0.00186  0.00364 | 0.0470  0.0183  0.03510.0316  0.0683  0.0349  0.0638  0.0638  0.0167  0.0364  0.0967  0.0338 |
| **Silva and others model** | | | | | | |
| CR1  CR2  CR3  US-HAD1  US-HAD2  US-HAD3  US-HAD4  US-HAD5  US-HAD6  US-HAD7  US-HAD8  US-HAD9 | 60  70  80  60  70  80  60  70  80  60  70  80 | a.0.035, b.-0.078  a.0.034, b.-0.011  a.0.060, b.-0.100  a.0.035, b.-0.079  a.0.037, b.-0.011  a.0.061, b.-0.015  a.0.035, b.-0.058  a.0.029, b.-0.008  a.0.065, b.-0.079  a.0.027, b.-0.011  a.0.022, b.0.039  a.0.058, b.-0.100 | 0.991  0.996  0.998  0.988  0.996  0.999  0.999  0.999  0.996  0.996  0.999  0.999 | 0.01  0.00  0.00  0.01  0.00  0.00  0.00  0.00  0.00  0.00  0.01  0.00 | 0.00082  0.00025  0.00030  0.00065  0.00096  0.00019  0.00035  0.00085  0.00045  0.00037  0.00096  0.00093 | 0.0262  0.0149  0.0357  0.0356  0.0961  0.0367  0.0961  0.0147  0.0852  0.0368  0.0751  0.0359 |
| **Aghbashlo and others mode** | | | | | | |
| CR1  CR2  CR3  US-HAD1  US-HAD2  US-HAD3  US-HAD4  US-HAD5  US-HAD6  US-HAD7  US-HAD8  US-HAD9 | 60  70  80  60  70  80  60  70  80  60  70  80 | k1.-17.072, k2.17.049  k1.0.959, k2.-0.991  k1.-34.931, k2.34.891  k1.14.225, k2.-14.248  k1.-10.475, k2.10.440  k1.-30.410, k2.30.377  k1.18.838, k2.-18.863  k1.21.969, k2.-21.997  k1.44.792, k2.-44.840  k1.-12.273, k2.12.248  k1.6.839, k2.-6.867  k1.-38.023, k2.37.985 | 0.973  0.996  0.984  0.966  0.996  0.952  0.987  0.998  0.990  0.996  0.994  0.984 | 0.01  0.00  0.00  0.02  0.00  0.00  0.03  0.11  0.00  0.00  0.01  0.00 | 0.00068  0.00034  0.00085  0.00037  0.00034  0.00039  0.00030  0.00078  0.00065  0.00037  0.00068  0.00037 | 0.0108  0.0037  0.0357  0.0963  0.0074  0.0258  0.0369  0.0257  0.0159  0.0035  0.0134  0.0357 |
| **Page Model** | | | | | | |
| CR1  CR2  CR3  US-HAD1  US-HAD2  US-HAD3  US-HAD4  US-HAD5  US-HAD6  US-HAD7  US-HAD8  US-HAD9 | 60  70  80  60  70  80  60  70  80  60  70  80 | k.0.009, n.1.245  k.0.030, n.1.026  k.0.017, n.1.250  k.0.006, n.1.351  k.0.030, n.1.051  k.0.005, n.1.566  k.0.010, n.1.234  k.0.024, n.0.036  k.0.024, n.1.218  k.0.022, n.1.028  k.0.044, n.0.882  k.0.016, n.1.272 | 0.986  0.996  0.995  0.989  0.996  0.999  0.999  0.999  0.996  0.996  0.999  0.997 | 0.02  0.00  0.00  0.01  0.00  0.00  0.00  0.00  0.00  0.00  0.00  0.00 | 0.00145  0.00025  0.00061  0.00018  0.00020  0.00039  0.00028  0.00019  0.00030  0.00034  0.00089  0.00073 | 0.0351  0.0150  0.02280.0367  0.0126  0.0069  0.0349  0.0937  0.0943  0.0761  0.0258  0.0437 |
| **Wang and Singh Model** | | | | | | |
| CR1  CR2  CR3  US-HAD1  US-HAD2  US-HAD3  US-HAD4  US-HAD5  US-HAD6  US-HAD7  US-HAD8  US-HAD9 | 60  70  80  60  70  80  60  70  80  60  70  80 | a.-0.018, b.8.68  a.-0.025, b.0.00  a.-0.032, b.0.00  a.-0.018, b.9.15  a.-0.027, b.0.00  a.-0.024, b.0.00  a.-0.020, b.0.00  a.-0.023, b.0.00  a.-0.037, b.0.00  a.-0.020, b.0.00  a.-0.022, b.0.00  a.-0.030, b.0.00 | 0.980  0.993  0.994  0.984  0.992  0.987  0.998  0.998  0.996  0.994  0.974  0.995 | 0.02  0.00  0.00  0.02  0.01  0.01  0.00  0.00  0.00  0.00  0.02  0.00 | 0.00163  0.00084  0.00083  0.00028  0.00035  0.00006  0.00018  0.00358  0.00156  0.00135  0.00896  0.00028 | 0.0036  0.0015  0.0120  0.0103  0.0037  0.0110  0.0108  0.0296  0.0134  0.0003  0.0137  0.0107 |
| **Newton** | | | | | | |
| CR1  CR2  CR3  US-HAD1  US-HAD2  US-HAD3  US-HAD4  US-HAD5  US-HAD6  US-HAD7  US-HAD8  US-HAD9 | 60  70  80  60  70  80  60  70  80  60  70  80 | k.0.023  k.0.033  k.0.040  k.0.023  k.0.036  k.0.033  k.0.025  k.0.028  k.0.048  k.0.025  k.0.028  k.0.039 | 0.973  0.996  0.984  0.966  0.996  0.952  0.987  0.998  0.987  0.996  0.994  0.984 | 0.04  0.00  0.01  0.04  0.00  0.03  0.01  0.02  0.00  0.03  0.01  0.00 | 0.00321  0.00037  0.00193  0.00034  0.00048  0.00288  0.00130  0.00016  0.00035  0.00079  0.00019  0.00028 | 0.0521  0.0182  0.0406  0.0583  0.0204  0.0672  0.0343  0.0134  0.0368  0.0535  0.0035  0.0029 |

CTL= Control; US-HAD= Ultrasound hot air drying. CR= Control; US-IR= Ultrasound infrared drying

**Supplementary Table S4**: Effect of US pretreatments with HAD and IR drying methods on the CIE surface color variables of sweet potatoes,

| **Sample codes** | **Temperature**  **^o^C** | **Frequency**  **kHz** | **ΔE** | **Hue** | **Croma** | **Yellowness** | **Whiteness** |
| --- | --- | --- | --- | --- | --- | --- | --- |
| **HAD** | | | | | | | |
| Fresh | -- | -- | -- | 79.05 | 40.52 | 69.10 | 55.75 |
| CTL1 | 60 | -- | 15.45k | 75.69c | 16.68l | 46.16b | 65.48k |
| CTL2 | 70 | -- | 17.88i | 75.06e | 18.65j | 41.29d | 67.39j |
| CTL3 | 80 | -- | 14.61l | 74.26g | 17.46k | 49.41a | 63.78l |
| US-HAD1 | 60 | 20 | 18.34h | 72.27j | 23.10e | 40.38f | 67.98g |
| US-HAD2 | 70 | 20 | 19.00g | 75.42d | 22.53f | 40.76e | 67.59i |
| US-HAD3 | 80 | 20 | 20.61e | 74.17h | 20.54h | 36.26h | 69.79c |
| US-HAD4 | 60 | 40 | 17.23j | 69.25l | 24.51c | 41.56c | 67.90h |
| US-HAD5 | 70 | 40 | 22.03d | 73.34 | 19.54i | 35.11i | 70.5a |
| US-HAD6 | 80 | 40 | 24.15b | 77.62a | 27.44a | 32.42k | 69.62d |
| US-HAD7 | 60 | 60 | 19.42f | 74.43f | 25.63b | 30.33l | 69.17f |
| US-HAD8 | 70 | 60 | 22.65c | 77.53b | 23.31d | 34.06j | 69.91b |
| US-HAD9 | 80 | 60 | 24.82a | 72.03k | 21.86g | 37.95g | 69.18e |
| **CIR drying** | | | | | | | |
| CR1 | 60 | -- | 12.1j | 76.54j | 13.44l | 52.65c | 62.79j |
| CR2 | 70 | -- | 27.77c | 79.01c | 14.69k | 28.58k | 59.66k |
| CR3 | 80 | -- | 11.43k | 76.12l | 14.86j | 51.44d | 53.32l |
| US-IR1 | 60 | 20 | 30.28a | 78.90 | 40.13a | 31.48j | 63.81e |
| US-IR2 | 70 | 20 | 19.04h | 77.41f | 29.54b | 52.98b | 63.06h |
| US-IR3 | 80 | 20 | 16.08i | 76.84h | 26.41d | 48.72e | 63.71f |
| US-IR4 | 60 | 40 | 27.28d | 76.83i | 26.25e | 36.67i | 63.05i |
| US-IR5 | 70 | 40 | 19.52g | 77.10g | 17.74i | 45.3f | 68.44a |
| US-IR6 | 80 | 40 | 20.84f | 77.74e | 23.61f | 38.94h | 67.47b |
| US-IR7 | 60 | 60 | 29.76b | 79.32b | 20.95h | 26.98l | 67.05c |
| US-IR8 | 70 | 60 | 8.297l | 79.37a | 29.43c | 73.6a | 63.39g |
| US-IR9 | 80 | 60 | 21.12e | 76.47k | 38.85a | 44.06g | 64.13d |
| Temperature (T) | -- | -- | <0.0001*** | <0.0001*** | <0.0001*** | <0.0001*** | <0.0001*** |
| Frequency (F) | -- | -- | <0.0001*** | <0.0001*** | <0.0001*** | <0.0001*** | <0.0001*** |
| T × F | -- | -- | <0.0001*** | <0.0001*** | <0.0001*** | <0.0001*** | <0.0001*** |

CTL= Control; US-HAD= Ultrasound hot air drying. CR= Control; US-IR= Ultrasound infrared drying

**Supplementary Tables References**

[1] A. El-Beltagy, G.R. Gamea, A.H.A. Essa, Solar drying characteristics of strawberry, J. Food Eng. 78 (2007) 456–464. https://doi.org/10.1016/j.jfoodeng.2005.10.015.

[2] E.O.M. Akoy, Experimental characterization and modeling of thin-layer drying of mango slices, Int. Food Res. J. 21 (2014) 1911–1917. https://pdfs.semanticscholar.org/eee4/569452a709c33a45b20e4a48a06d6f8803cf.pdf (accessed December 29, 2018).

[3] N. Hashim, O. Daniel, E. Rahaman, A Preliminary Study: Kinetic Model of Drying Process of Pumpkins (Cucurbita Moschata) in a Convective Hot Air Dryer, Agric. Agric. Sci. Procedia. 2 (2014) 345–352. https://doi.org/10.1016/j.aaspro.2014.11.048.

[4] M.S. Zenoozian, H. Feng, S.M.A. Razavi, F. Shahidi, H.R. Pourreza, Image analysis and dynamic modeling of thin-layer drying of osmotically dehydrated pumpkin, J. Food Process. Preserv. 32 (2008) 88–102. https://doi.org/10.1111/j.1745-4549.2007.00167.x.

[5] K. Kulwinder, S. A.K., Drying kinetics and quality characteristics of beetroot slices under hot air followed by microwave finish drying, African J. Agric. Res. 9 (2014) 1036–1044. https://doi.org/10.5897/AJAR2013.

[6] M. Campus, A.- Bellevue, Mathematical modeling of thin layer drying kinetics of apples slices, Int. Food Res. J. 19 (2006) 1949–1958. https://doi.org/10.1051/IUFoST.

[7] K. Sacilik, Effect of drying methods on thin-layer drying characteristics of hull-less seed pumpkin (Cucurbita pepo L.), J. Food Eng. 79 (2007) 23–30. https://doi.org/10.1016/j.jfoodeng.2006.01.023.

[8] C.L. Hii, C.L. Law, M. Cloke, Modeling using a new thin layer drying model and product quality of cocoa, J. Food Eng. 90 (2009) 191–198. https://doi.org/10.1016/j.jfoodeng.2008.06.022.

[9] I. Stoilova, A. Krastanov, A. Stoyanova, P. Denev, S. Gargova, R. Pérez-Rosés, E. Risco, R. Vila, P. Peñalver, S. Cañigueral, Y. Shukla, M. Singh, R.P. Jakribettu, R. Boloor, H.P. Bhat, A. Thaliath, R. Haniadka, M.P. Rai, T. George, M.S. Baliga, A. Jelled, ngela Fernandes, L. Barros, H. Chahdoura, L. Achour, I.C.F.R. Ferreira, H. Ben Cheikh, Y. Li, Y. Hong, Y. Han, Y. Wang, L. Xia, K. An, D. Zhao, Z. Wang, J. Wu, Y. Xu, G. Xiao, ??zlem Akt??rk G??m????ay, A.A. Borazan, N. Ercal, O. Demirkol, R.B. Semwal, D.K. Semwal, S. Combrinck, A.M. Viljoen, B.H. Ali, G. Blunden, M.O. Tanira, A. Nemmar, Y. Zhu, P. Wang, Y. Zhao, C. Yang, A. Clark, T. Leung, X. Chen, S. Sang, Z. Yang, D. Zhang, Y. Zhang, M. Wu, H. Liu, X. Han, Q. Zheng, Y. Huang, C. Chen, L. Zhang, D. Yan, Y. Zhao, X. Xiao, A. Ghasemzadeh, H. Jaafar, A. Rahmat, Comparison of different drying methods on Chinese ginger (Zingiber officinale Roscoe): Changes in volatiles, chemical profile, antioxidant properties, and microstructure, Food Chem. 77 (2016) 4716–4724. https://doi.org/10.1016/j.phytochem.2015.07.012.

[10] P.L. Gan, P.E. Poh, Investigation on the Effect of Shapes on the Drying Kinetics and Sensory Evaluation Study of Dried Jackfruit, Int. J. Sci. Eng. 7 (2014) 193–198. https://doi.org/10.12777/ijse.7.2.193-198.

[11] İ. Doymaz, S. Karasu, M. Baslar, Effects of infrared heating on drying kinetics, antioxidant activity, phenolic content, and color of jujube fruit, J. Food Meas. Charact. 10 (2016) 283–291. https://doi.org/10.1007/s11694-016-9305-4.

[12] A.O. Omolola, A.I.O. Jideani, P.F. Kapila, Modeling microwave drying kinetics and moisture diffusivity of mabonde banana variety, Int. J. Agric. Biol. Eng. 7 (2014) 107–113. https://doi.org/10.3965/j.ijabe.20140706.013.

[13] W.P. da Silva, C.M.D.P.S. e Silva, F.J.A. Gama, J.P. Gomes, Mathematical models to describe thin-layer drying and to determine drying rate of whole bananas, J. Saudi Soc. Agric. Sci. 13 (2014) 67–74. https://doi.org/10.1016/j.jssas.2013.01.003.
